# Suppression of ADP-ribosylation reversal triggers cell vulnerability to alkylating agents

**DOI:** 10.1016/j.neo.2024.101092

**Published:** 2024-11-29

**Authors:** Rocco Caggiano, Evgeniia Prokhorova, Lena Duma, Kira Schützenhofer, Raffaella Lauro, Giuliana Catara, Rosa Marina Melillo, Angela Celetti, Rebecca Smith, S John Weroha, Scott H Kaufmann, Ivan Ahel, Luca Palazzo

**Affiliations:** a Institute of Experimental Endocrinology and Oncology, National Research Council of Italy, Naples, Italy; b Sir William Dunn School of Pathology, University of Oxford, Oxford, United Kingdom; c Department of Molecular Medicine and Medical Biotechnology, University of Naples “Federico II”, Naples, Italy; d Institute of Biochemistry and Cell Biology, National Research Council of Italy, Naples, Italy; e Department of Oncology, Mayo Clinic, Rochester, Minnesota, United States

**Keywords:** DNA damage, ADP-ribosylation, ADP-ribosyl hydrolases, ARH3, PARG inhibitor, Alkylating drugs, PARP inhibitor, Cancer

## Abstract

The ADP-ribosyl hydrolases PARG and ARH3 counteract PARP enzymatic activity by removing ADP-ribosylation. PARG and ARH3 activities have a synthetic lethal effect; however, the specific molecular mechanisms underlying this response remain unknown. Here, we show that the PARG and ARH3 synthetic lethality is enhanced further in the presence of DNA alkylating agents, suggesting that the inability to revert ADP-ribosylation primarily affects the repair of alkylated DNA bases. *ARH3* knockout cells, treated with PARG inhibitor and alkylating genotoxins, accumulated single-stranded DNA and DNA damage, resulting in G2/M cell cycle arrest and apoptosis. Furthermore, we reveal a reduction in PARP1/PARP2 levels in *ARH3*-deficient cells treated with PARG inhibitor due to excessive ADP-ribosylation, which may contribute to alkylating agents’ vulnerability. Collectively, these results uncover the potential of targeting ADP-ribosyl hydrolases in combination with alkylating agents for cancer therapy and provide insights into the mechanisms underlying the synthetic lethal effect.

## Highlights

• ARH3 deficiency results in increased sensitivity to PARGi in ovarian cancer cells.
• ARH3 deficiency results in resistance to PARPi in ovarian cancer cells.
• ARH3 and PARG loss induces alkylating agent sensitivity.
• Combining ARH3 and PARG inhibition with TMZ may represent a novel therapeutic strategy.

## Graphical abstract

**Figure:**
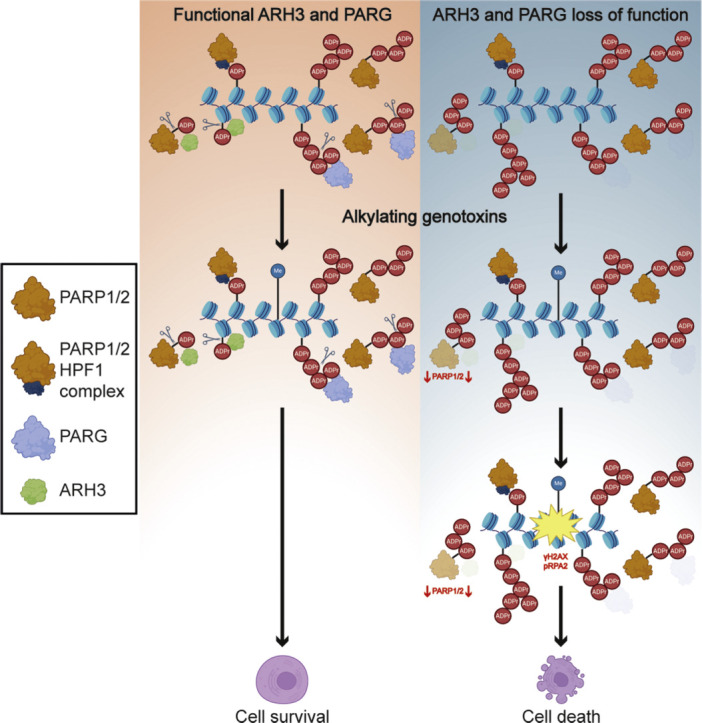

**List of abbreviations**
***ARH3***: ADP-ribosyl hydrolase 3
***BER***: Base Excision Repair
***CPT***: camptothecin
***DSBs***: double-strand breaks
***5-FU***: 5-fluorouracil
***HPF1***: Histone PARylation Factor 1
***HRR***: homologous recombination repair
***HU***: hydroxyurea
***KO***: knockout
***MAR***: mono(ADP-ribose)
***MARylation***: mono(ADP-ribosyl)ation
***MMS***: methyl methanesulfonate
**NER**: Nucleotide Excision Repair
***PAR***: poly(ADP-ribose)
***PARG***: Poly(ADP-ribose) glycohydrolase
***PARGi***: PARG inhibitor
***PARP***: Poly(ADP-ribose) polymerase
***PARPis***: PARP inhibitors
***PARylation***: poly(ADP-ribosyl)ation
***pRPA2***: phospho-Ser8-RPA2
***Ser-ADPr***: serine ADP-ribosylation
***SSBs***: single-strand breaks
***TMZ***: temozolomide
***WT***: wild-type

## Introduction

Poly(ADP-ribose) polymerases (PARPs) transfer single or multiple units of ADP-ribose from NAD^+^ to target proteins and nucleic acids, a process called ADP-ribosylation [1,2]. PARP1 and PARP2 (PARP1/2) play essential roles in maintaining genome stability. Mechanistically, DNA breaks activate PARP1/2, whose poly(ADP-ribosyl)ation (PARylation) represents one of the earliest events in response to single-strand breaks (SSBs), double-strand breaks (DSBs), and disturbed replication forks [1,[3], [4], [5]]. By ADP-ribosylating protein substrates, PARP1/2 trigger the recruitment and regulation of DNA repair enzymes and chromatin remodellers, thereby facilitating DNA repair [4,6]. Accordingly, PARP inhibitors (PARPis) represent an established treatment strategy for ovarian, breast, pancreatic, prostate, and small-cell lung cancers harboring defects in DNA repair pathways [[7], [8], [9]].

After DNA damage, large amounts of serine-linked ADP-ribose (Ser-ADPr) and chains of ADP-ribose are formed [[10], [11], [12], [13]]. To synthesize Ser-ADPr, the accessory factor HPF1 forms a complex with PARP1 or PARP2 to catalyze the initial serine mono-ADP-ribose adduct, which can be further extended by PARP1/2 alone into poly(ADP-ribose) (PAR) chains [11,[14], [15], [16], [17], [18], [19]]. Without HPF1, activated PARP1/2 can also attach ADP-ribose to other amino acids in proteins, such as glutamate, or target nucleic acids [[20], [21], [22], [23], [24], [25]].

ADP-ribosyl hydrolases counteract PARP enzymatic activity to ensure that protein ADP-ribosylation is short-lived. Reversal of Ser-ADPr involves two steps: *i)* cleavage of PAR chains by poly(ADP-ribose) glycohydrolase (PARG) into mono-ADP-ribosylation (MAR) [26,27]; and *ii)* removal of ADP-ribose covalently linked to serine by ADP-ribosyl hydrolase 3 (ARH3), the only known human enzyme able to reverse serine-linked MARylation [11,15,28,29]. In contrast to PARG, whose activity slows as PAR chains shorten [30], ARH3 can inefficiently hydrolyze PAR chains and cannot cleave PAR branches [29]. In addition, ARH3 and PARG show some activity against PAR linked to other amino acids, such as glutamate [[31], [32], [33]].

Targeting reversal of protein ADP-ribosylation has been identified as a promising approach for treating cancers, especially those resistant to PARPis. The loss of ARH3 or PARG results in PARPi resistance in cells [19,[34], [35], [36]]; moreover, depletion or inhibition of either ARH3 or PARG creates a dependence on the remaining intact enzyme and increases susceptibility to its inhibition. In particular, *ARH3* knockout (KO) sensitizes cells to PARG inhibitor (PARGi) due to persistent high levels of PARylation, ultimately causing cell death [19]. Notably, a PARGi has entered Phase I clinical trials (ClinicalTrials.gov ID: NCT05787587) [9,37], providing the potential means for treating *ARH3*-mutated tumors. However, the molecular mechanisms by which PARGis kill *ARH3*-deficient cells remain incompletely understood.

Here, we explore the potential impact of the combined therapeutic approach involving ARH3 and PARG inhibition on cancer cells, focusing on ovarian cancer cells because of the crucial role of PARylation on ovarian cancer resistance to chemotherapeutics [9,35,[38], [39], [40], [41], [42], [43]]. Our research shows that combined *ARH3* gene interruption and PARG inhibition sensitizes cancer cells to alkylating genotoxins, providing new insights into the role of protein ADP-de-ribosylation in DNA repair after alkylation-induced DNA damage and suggesting new potential drug combinations for therapy.

## Materials and methods

### Cell culture

Human osteosarcoma U2OS (ATCC HTB-96) cells were grown in Dulbecco′s Modified Eagle′s Medium (DMEM; Merck Millipore) with 4,500 mg/L glucose, L-glutamine, sodium pyruvate, and sodium bicarbonate, supplemented with 10% heat-inactivated Fetal Bovine Serum (FBS; Merck Millipore). Human ovarian adenocarcinoma PEO1 (Fergus Couch, Mayo Clinic) COV362 and OVCAR8 cells [44] were cultured in DMEM (COV362) or RPMI-1640 (PEO1 and OVCAR8) supplemented with 10% (COV362 and PEO1) or 20% (OVCAR8) FBS. All cell lines were cultured at 37°C with 5% CO_2_ and authenticated by short tandem repeat analysis.

### Generation of cell lines

Gene KO cell lines were generated using the CRISPR/Cas9 technology, as previously described [11,14,15,17,19], using the following primers: sgRNA #210 (GCGCTGCTCGGGGACTGCGT) and sgRNA #212 (GGGCGAGACGTCTATAAGGC) for the generation of *ARH3* gene KO, sgRNA #1(CCACCTCAACGTCAGGGTG) and sgRNA #2 (TGGGTTCTCTGAGCTTCGT) for the generation of *PARP1* gene KO cell lines, sgRNA #1(CAGCAGAATTCCCCGATCCG) and sgRNA #2 (TCGGCGGTGGCGGGAAGCGC) for the generation of *HPF1* gene KO cell lines. Primers were cloned into pX459(1.1). 48 hours after transfection with empty pX459 or co-transfection with specific sgRNA pairs (1:1 ratio) using TransIT-LT1 Transfection Reagent (Mirus Bio), cells were selected with 2 μg/ml Puromycin (InvivoGen) for 72 hours and seeded on 96-well plates at 0.4 cells/well to propagate single colonies. Individual clones were then screened by western blotting. Flag western blotting was used to exclude clones expressing Cas9. To introduce untagged ARH3 into *ARH3^-/-^* PEO1, the ARH3 cDNA was cloned into pLX304 (Addgene #25890) using LR Clonase II enzyme mix (Life Technologies). Lentivirus particles were produced by co-transfecting pLX304-ARH3 WT or pLX304-ARH3 D77/78N along with packaging plasmids pCMV-VSV-G and pCMV-dR8.2 dvpr (Addgene) into 293T cells using FuGENE (Promega). The supernatant was collected 24 hours after transfection, filtered, and used to infect cells. After 48 h, stable integrants were selected with 6 μg/mL Blasticidin (Invitrogen) for 8 days. ARH3 expression levels were analyzed by immunoblotting. *ARH3* KO U2OS cells complemented with wild-type ARH3 (ARH3 WT) or catalytically inactive D77/78N mutant were described previously [19].

### Cell proliferation and survival assays

Cell viability was assessed using CellTiter 96 AQueous One Solution (Promega; Cat# PRO-G3580) per the supplier's instructions. Cells were seeded in 96-well plates at 3,000 cells/well and incubated under the specified conditions for 24, 48, or 72 hours. For colony formation assays, cells were plated in 6-well plates (700 cells/well for U2OS cells) or 24-well plates (300 cells/well for U2OS cells, 150 cells/well for PEO1 cells, 300 cells/well for COV362 cells, 50 cells/well for OVCAR8 cells), incubated under the specified conditions for 11 days, fixed, stained with 0.5% crystal violet in 25% methanol for 30 minutes, washed with water, and air-dried. Quantification was carried out using ImageJ or ICY software. The surviving fraction at each dose was calculated after normalizing to the plating efficiency of untreated samples. Each experiment was carried out in technical triplicates and repeated independently three times.

### Cell lysis, fractionation, and western blotting

Cells were lysed with Triton X-100 lysis buffer (50 mM Tris-HCl pH 8.0, 100 mM NaCl, 1% Triton X-100) supplemented with 5 mM MgCl_2_, protease (Merck Millipore, Cat. 4906837001) and phosphatase inhibitors (Merck Millipore, Cat. 11873580001), olaparib (1 μM), PARGi PDD00017273 (1 μM) at 4°C. The lysates were incubated with 0.1% Benzonase (Merck Millipore, Cat. E1014) for 30 minutes at 4°C, centrifuged at 14,000 rpm for 15 minutes, and the supernatants were collected. As significant amounts of histone proteins could be lost after centrifugation, both supernatant (soluble) and pellet (insoluble) fractions were used as controls for fractionation experiments. Total cell lysates were prepared for some experiments as above but without the centrifugation step. For enzymatic reactions with recombinant PARG, 10 μg of the total cell extract was resuspended in 10% glycerol solution supplemented with 25 mM Tris-HCl pH 8.0, 150 mM NaCl, 1 mM DTT, and incubated or not with 10 μM recombinant PARG for 30 minutes at 37°C. A commercial kit (Thermo Fisher Scientific, cat. 78840) was used according to the supplier's instructions for the subcellular protein fractionation. Protein concentrations were analyzed using Bradford Protein Assay (Bio-Rad). Proteins were boiled in 1x NuPAGE LDS sample buffer (Life Technologies) with TCEP or DTT (Sigma), resolved on NuPAGE 4%–12% Bis-Tris gels (Life Technologies), and transferred to nitrocellulose membranes (Bio-Rad) using Trans-Blot Turbo Transfer System (Bio-Rad). Membranes were blocked in PBS buffer with 0.1% Tween 20 and 5% non-fat dried milk for 1-h at room temperature and incubated overnight with primary antibodies (1:1,000 unless stated otherwise) at 4°C, followed by 1hour incubation with peroxidase-conjugated secondary anti-mouse (Agilent, cat. P0447, 1:3,000) or anti-rabbit (Agilent, cat. P0399, 1:3,000) antibody at room temperature. Blots were developed using ECL (Thermo Fisher Scientific) and analyzed by exposure to X-ray films.

### Antibodies

In this study, the following antibodies were used: anti-poly/mono ADPr (rabbit monoclonal), Cell Signaling, Cat. 83732; anti-H2AX (rabbit polyclonal), Cell Signaling, Cat. 2595; anti-PARP1 (rabbit monoclonal), Abcam, Cat. ab32138; anti-PARP1 (mouse monoclonal); BD Biosciences, Cat. 556494; anti-γH2AX (mouse monoclonal), Cell Signaling, Cat. D7T2V; anti-α-tubulin (mouse monoclonal), Sigma-Aldrich, Cat. T6074; anti-β-tubulin (rabbit polyclonal), Abcam, Cat. ab6046; anti-Phospho-RPA32/RPA2 (Ser8) (rabbit polyclonal), Cell Signaling, Cat. 54762; anti-RPA32 p-S4/8 (rabbit polyclonal), Bethyl, Cat. A300-245A; anti-RPA32 (rabbit polyclonal), Bethyl, Cat. A300-244A; RPA32/RPA2 (rabbit polyclonal) Cell Signaling, Cat. 52448; anti-PAN-ADP-RIBOSE binding reagent, Merck Millipore, Cat. MABE1016; anti-Poly(ADP-ribose) (rabbit polyclonal), Enzo Life Sciences, Cat. ALX-210-890A-0100; anti-ADPRHL2 (rabbit monoclonal), Sigma-Aldrich, Cat. HPA027104; anti-histone H3 (rabbit polyclonal), Millipore, Cat. 07–690; anti-p53 (mouse monoclonal), Santa Cruz, Cat. sc-126; anti-p53 K370ac, Cell Signaling; anti-p53 K382ac, Cell Signaling, Cat. 2525; anti-Caspase 3 (rabbit monoclonal), Cell Signaling, Cat. 14220; anti-Caspase 7 (rabbit monoclonal); Cell Signaling, Cat. 12827; anti-HPF1/C4orf27 (rabbit polyclonal), NovusBio; Cat. NBP1-93973; anti-H2A (rabbit polyclonal), Abcam, Cat. ab18255; anti-PARP2 (mouse monoclonal), Millipore, Cat. MABE18; anti-lamin A (rabbit polyclonal), Abcam, Cat. ab26300;

### Chemicals and critical commercial kits

PDD00017273 (PARGi), MCE, Cat. HY-108360; olaparib, MCE, Cat. HY-10162; Methyl methanesulfonate (MMS), Merck Millipore, Cat. 129925; Crystal violet, Merck Millipore, Cat. C0775; Puromycin, Invivogen, Cat. ANT-PR-1; Blasticidin, Invivogen, Cat. ANT-PR-1; NuPAGE LDS sample buffer, Life Technologies, Cat. NP0008; 5-Fluorouracil (5-FU), Sigma, Cat. F6627; Camptothecin (CPT), Selleckchem, Cat. S1288; Cisplatin, Merck Millipore, Cat. P4394; Paclitaxel, Abcam, Cat. ab120143; Nicotinamide Riboside (NR), Cayman Chemical, Cat. 23132; Nicotinic Acid (NA), Selleckchem, Cat. S1744; Nicotinamide (NAM), Tocris, Cat. 4106; Hydroxyurea, Merck Millipore, Cat. 1016970001; Temozolomide (TMZ), MCE, Cat. HY-17364; DAPI, Merck Millipore, Cat. D9542; TCEP, Merck Millipore, Cat. 646547; Trichostatin A, Merck Millipore, Cat. T8552; TransIT-LT1 Transfection Reagent, Mirus Bio, Cat. MIR 2300;

### Flow cytometry

Cells were seeded in 6-well plates and treated as indicated. For Annexin V-DAPI staining, cells were harvested by trypsinization and labeled with Annexin V-FITC (Life Technologies) and DAPI (0.1 μg/mL; Merck Millipore) in 1X Annexin V binding buffer (Life Technologies) following the manufacturer's instructions. Cells were analyzed immediately after staining on a Cytek DxP8 (Becton Dickinson). For cell cycle analysis, cells were incubated with 10 μM EdU for 1 h at the end of treatment, harvested by trypsinization, and labeled using the Click-iT Plus EdU Alexa Fluor 647 Flow Cytometry Assay Kit (Life Technologies; Cat. C10419) according to the manufacturer's instructions. For DAPI staining, cell pellets were resuspended in 0.1 μg/mL DAPI solution in PBS and incubated and protected from light for 10 min. Cells were washed in PBS and analyzed immediately after staining on Cytoflex LX (Beckman Coulter). 10,000 events per sample were recorded. Post-acquisition analysis was performed using FlowJo software (BD Biosciences).

### Immunofluorescence and confocal microscopy

Cells were seeded on glass coverslips and incubated under the specified conditions. The cells were rinsed with PBS and then treated with 0.2% Triton X-100/PBS solution containing 1 μM olaparib and 1 μM PARGi for 5 minutes for pre-extraction. After the PBS rinse, the cells were fixed with 4% paraformaldehyde (PFA, Merck Millipore) supplemented with 1 μM olaparib and 1 μM PARGi for 15 minutes, followed by a PBS wash and permeabilization with 0.2% Triton X-100/PBS for 10 minutes. Subsequently, the cells were blocked with 3% BSA/PBS for 30 minutes. Next, the cells were incubated with primary antibodies (Poly/Mono-ADP Ribose, Cell Signaling Technology, Cat. 83732, 1:500; anti-phospho-Ser8-RPA32/RPA2, Cell Signaling Technology, Cat. 54762, 1:500; anti-γH2AX, Cell Signaling Technology, Cat. D7T2V, 1:500) in 3% BSA/PBS for 1 hour at room temperature. This was followed by incubation with the secondary antibodies: Alexa Fluor 488 anti-rabbit IgG (Life Technologies, Cat. A32731) or Alexa Fluor 594 anti-mouse IgG (Life Technologies, Cat. A32744) for 1 hour, supplemented with 0.1 μg/mL DAPI (4,6-diamidino-2-phenylindole, Merck Millipore). After three PBS washes, the coverslips were mounted onto glass slides with Mowiol 4-88 (Merck Millipore). Images were captured using an inverted and motorized microscope (Axio Observer Z.1) with a 63 × /1.4 Plan Apochromat or a 20 × /0.5 EC Plan Neofluar objective. Confocal imaging was enabled by the attached laser scanning unit (LSM 700 4 × pigtailed laser 405–488–555–639; Zeiss, Jena, Germany). To improve the readability of γH2AX and phospho-Ser8-RPA2 foci, equal adjustments were applied to the fluorescence of all images using the ZEISS Zen Microscopy software after image capture. Specifically, the parameters for each channel were set from the image display bar as follows: red (black, 0, white, 30), green (black, 0, white, 90), and blue (black, 0, white, 160). Processing was applied to the entire images, uniformly for all samples. The number of γH2AX and phospho-Ser8-RPA2 foci was quantified using the Find Maxima tool, applying variable prominence values depending on the experiment in ImageJ (FiJi). Analysis was conducted on 50 single cells for each condition. The numbers of positive foci obtained from the software quantification were verified by visual inspection.

### Quantification and statistical analysis

Experiments in this study were performed in biological triplicates. Prism 10 (GraphPad) was used for statistical analysis, where ∗*p* < 0.05, ∗∗*p* < 0.01, ∗∗∗*p* < 0.001. Details of statistical analyses are described in the figure legends.

### Software

The software used in this work included Zen (Zeiss), ImageJ (NIH), Prism 10 (GraphPad), ICY (Institute Pasteur), Illustrator and Photoshop (Adobe), Word and Excel (Microsoft). The graphical abstract was created with bioRender.com.

## Results

### ARH3 loss impacts cancer cell response to PARPi and PARGi

**Fig. 1:**
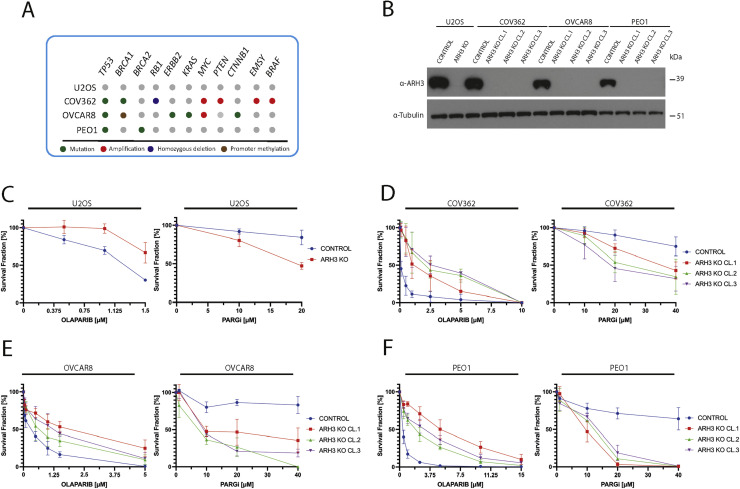
ARH3 loss impacts cancer cell response to PARPi and PARGi. (A) Schematic representation of the main gene mutation profile of the cell lines employed in this study. (B) Representative western blotting analysis of ARH3 protein levels in total cell lysates extracted from control and independent clones of *ARH3* KO U2OS, COV362, OVCAR8, and PEO1 cell lines. α-tubulin served as loading controls. (C-F) Survival fraction of cell colony formation assay performed in control and *ARH3* KO cells. U2OS, COV362, OVCAR8, and PEO1 cells were treated with olaparib and PARGi used at the indicated concentrations. Experiments were performed in biological and technical triplicates.

The regulation of PARylation is critical in ovarian cancer biology. PARPis are widely used in clinical treatment, and PARG inhibitors show promising results *in vitro* for high-grade serous ovarian cancer cells. Thus, we decided to assess the impact of *ARH3* knockout (KO) in ovarian cancer cell lines with different genetic features, including homozygous *BRCA1* mutant COV362 [45], *BRCA2* mutant PEO1 [46], which are deficient for homologous recombination repair (HRR), and *BRCA1/BRCA2* wild-type OVCAR8 that are HRR-competent despite the hypermethylation of two alleles of the three harbored by these cells [45,47,48] (Fig. 1A and 1B). All of these lines display TP53 inactivation, which is an almost universal feature of high-grade serous ovarian cancer. Previously described parental and *ARH3* KO U2OS osteosarcoma cells [19] served as controls for these studies. As already shown in U2OS cells, *ARH3* KO diminished the sensitivity of the ovarian cancer cell lines to the PARPi olaparib (Fig. 1C-F, left panels, and Figs. S1A, S1C, S1E, S1G). In contrast, *ARH3* KO enhanced sensitivity to the PARGi PDD00017273 (Fig. 1C-F, right panels, and Figures S1B, S1D, S1F, S1H) regardless of *BRCA1/2* status. Notably, all three independent clones of *ARH3* KO PEO1 cells formed smaller colonies in the long-term survival assays compared with the parental control for reasons currently unknown (Fig. S1).

### Methyl methanesulfonate contributes to enhanced PARGi sensitivity of ARH3 KO cells

**Fig. 2:**
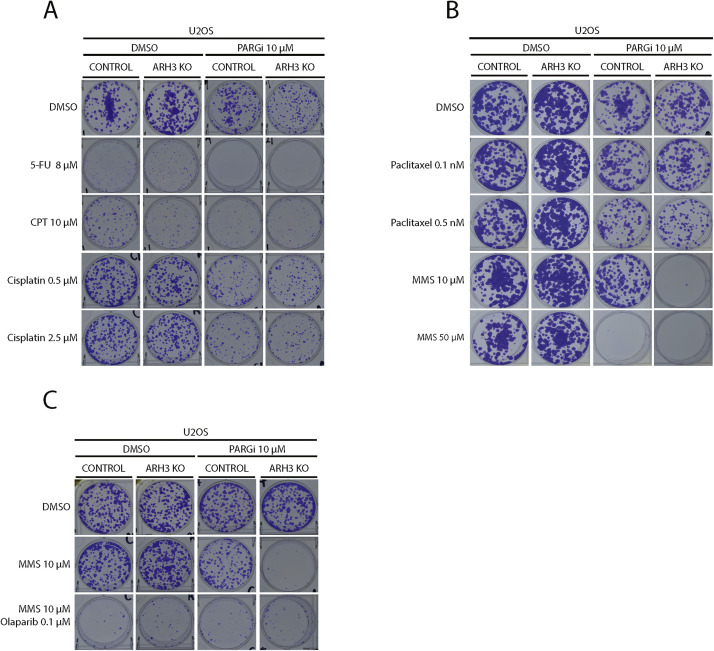
Methyl methanesulfonate contributes to enhanced PARGi sensitivity of *ARH3* KO cells. (A-B) Representative images of colony formation assays carried out in control and *ARH3* KO U2OS cells. As indicated, the cells were treated with or without 10 μM PARGi in combination with DMSO or genotoxins used at the indicated concentrations. (C) Representative images of colony formation assays carried out in control and *ARH3* KO U2OS cells. As indicated, the cells were treated with or without PARGi in combination with DMSO or MMS or a combination of MMS and olaparib used at the indicated concentrations. Each experiment shown in this figure was conducted in biological and technical triplicates.

To identify molecular pathways responsible for ARH3-dependent phenotypic outcomes, we conducted a small-scale drug screen in U2OS cells. To search for mechanisms that contribute to the synthetic lethality of *ARH3* loss and PARG inhibition, we compared the survival of control and *ARH3* KO U2OS cells, with and without PARGi treatment, when exposed to the thymidylate synthase inhibitor 5-fluorouracil (5-FU) [49], the topoisomerase I inhibitor camptothecin (CPT) [50], the purine crosslinking agent cisplatin [51] and the microtubule-directed agent paclitaxel [52,53]. Loss of ARH3 function did not significantly affect cell death when PARGi was combined with any of these agents (Fig. 2A and B).

In contrast, a different picture emerged when we examined the effect of combining PARGi with methyl methanesulfonate (MMS), an alkylating agent that transfers methyl groups to nucleophilic sites on DNA bases to form O6-methylguanine, N7-methylguanine, and N3-methyladenine [54,55], which are well known causes of SSBs and DSBs at stalled replication forks [4,55]. *ARH3* KO alone did not improve cell sensitivity to MMS (Fig. 2B). Strikingly, ARH3 loss markedly sensitized cells to the MMS/PARGi combination that had no impact on *ARH3* wild-type cells (Fig. 2B, 10 μM MMS). Notably, 5-fold higher MMS concentrations were necessary to kill control U2OS cells in the presence of PARGi (Fig. 2B, 50 μM MMS, and Fig. S2A). Consistently, MMS treatment activated PARP signaling, leading to a significant accumulation of ADP-ribosylated proteins over time in *ARH3* KO U2OS but not in control cells (Fig. S2B). Next, we examined the impact of PARP inhibition in this context. Low concentrations of olaparib (0.1 µM) not only sensitized both control and *ARH3* KO cells to MMS by itself (Fig. 2C) in line with the central role of PARP1/2 in repairing SSBs generated by Base Excision Repair (BER) following DNA alkylation [56], but also reduced the heightened sensitivity of *ARH3* KO U2OS to MMS due to less PAR production. By contrast, at a concentration of 0.1 µM, olaparib alone did not affect cell survival (Fig. S2C). Collectively, these results suggest that the repair of alkylated purines is one of the main cellular processes affected by complete inhibition of PAR degradation.

### Role of ARH3 catalytic activity in sensitization to MMS/PARGi combination

**Fig. 3:**
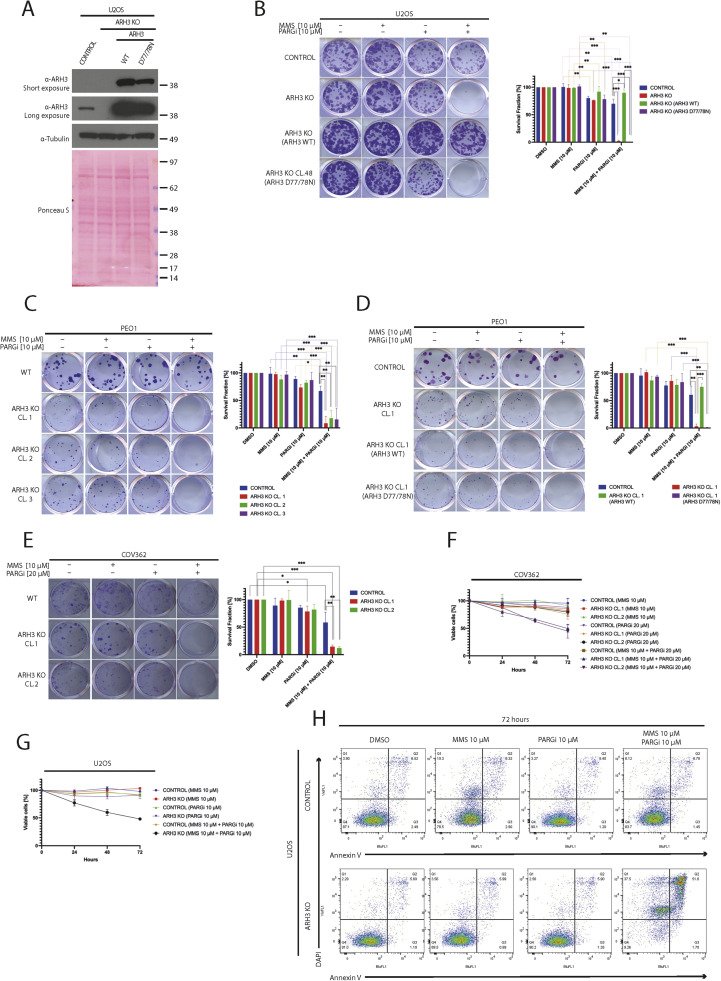
The combination of ARH3 KO and PARGi sensitizes diverse cancer cell lines to MMS through the loss of ARH3 catalytic activity. (A) Representative western blotting analysis of ARH3 protein in parental and *ARH3* KO U2OS cells, as well as in *ARH3* KO U2OS cells, complemented either wild-type ARH3 (ARH3 WT) or catalytically inactive ARH3 D77/78N through stable overexpression. α-tubulin and Ponceau S served as loading controls. (B) Representative images (left panel) and relative quantification with statistics (right panel) of colony formation assays conducted in parental, *ARH3* KO U2OS cells, *ARH3* KO U2OS cells complemented with either wild-type ARH3 (ARH3 WT) or catalytically inactive ARH3 D77/78N treated with DMSO, MMS, PARGi, or a combination of MMS and PARGi at the indicated concentrations. (C) Representative images (left panel) and relative quantification (right panel) of colony formation assays conducted in parental and independent clones of *ARH3* KO PEO1 cells treated as indicated. (D) Representative images (left panel) and relative quantification (right panel) of colony formation assays conducted in parental and *ARH3* KO PEO1 cells complemented with either wild-type ARH3 (ARH3 WT) or catalytically inactive ARH3 D77/78N treated as indicated. (E) Representative images (left panel) and relative quantification (right panel) of colony formation assays conducted in parental and independent clones of *ARH3* KO COV362 cells treated as indicated. (F-G) The short-term cell viability of parental and *ARH3* KO COV362 (F) and U2OS (G) cells was measured using the metabolic CellTiter 96® AQueous One Solution assay as cells were treated with DMSO, MMS, PARGi, or a combination of MMS and PARGi at the indicated concentrations for 24, 48, and 72 hours. (H) Representative scatterplots of flow cytometry analysis for Annexin V-DAPI stained cells after 72 hours of exposure of parental or *ARH3* KO U2OS cells as indicated. Each experiment shown in this figure was conducted in biological and technical triplicates. Quantification data are shown as mean ± SD. Statistical significance was evaluated by using a 2-tailed Student's t-test (∗*p* < 0.05, ∗∗*p* < 0.01, and ∗∗∗*p* < 0.001).

To assess whether sensitization to the MMS/PARGi combination was due to a loss of ARH3 catalytic activity versus a loss of other ARH3 functions, we introduced either wild-type ARH3 or catalytically dead ARH3 D77/78N into *ARH3* KO U2OS cells (Fig. 3A) [57]. Wild-type ARH3, but not the ARH3 D77/D78N mutant, restored resistance to MMS in the presence of PARGi, indicating that this effect requires ARH3 catalytic activity (Fig. 3B).

To confirm that the sensitivity to MMS and PARGi was not unique to U2OS cells, we examined control and *ARH3* KO PEO1 cells (Fig. 3C). All *ARH3* KO PEO1 clones, but not the parental cells, were susceptible to the combination of MMS and PARGi. Moreover, *ARH3* KO PEO1 cells re-expressing wild-type ARH3 or catalytically dead ARH3 D77/78N (Fig. S3A) behaved like corresponding U2OS cells (Fig. 3D and Figures S3B and S3C). Similar results were observed in COV362, where *ARH3* KO also conferred sensitivity to the MMS/PARGi combination (Fig. 3E). These results suggested that, regardless of *BRCA1/2* proficiency, inhibition of ADP-ribosylation hydrolysis rendered cells sensitive to MMS.

In further studies, the PARGi/MMS combination caused early cell death in *ARH3* KO cells, leading to a more than 50% decrease in cell viability within 72 hours as measured using MTT assays (Fig. 3F and G, and Figures S3D and S3E). The metabolic cell measurements were confirmed by Annexin V staining using flow cytometry (Fig. 3H). Collectively, these results support the hypothesis that combining ARH3 suppression with PARG inhibition sensitizes cells with a wide range of repair proficiencies to the alkylating agent MMS, leading to apoptosis.

### ARH3 loss and PARG inhibition also sensitize cancer cells to temozolomide

**Fig. 4:**
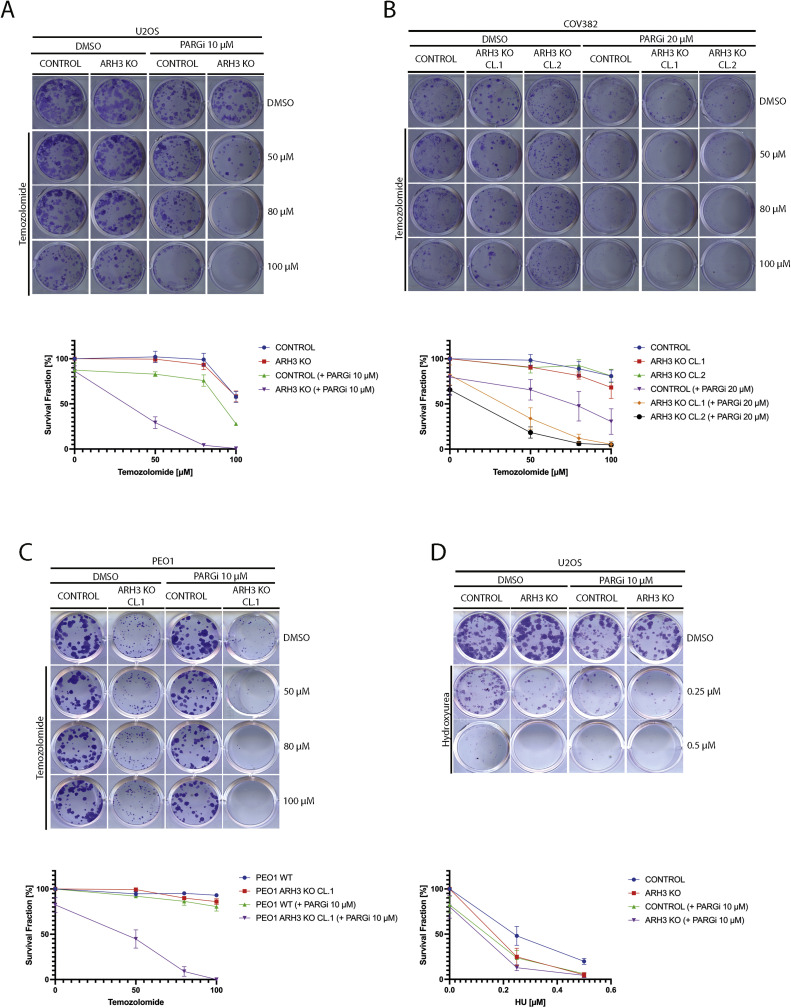
*ARH3* KO and PARG inhibition sensitize cancer cell lines to temozolomide. (A) Representative images (upper panel) and survival fraction (lower panel) from colony formation assay carried out in parental and *ARH3* KO U2OS cells treated with or without PARGi in combination with DMSO or temozolomide at the indicated concentrations. (B) Representative images (upper panel) and survival fraction (lower panel) from colony formation assay carried out in parental and two independent clones of *ARH3* KO COV362 cells treated with or without PARGi in combination with DMSO or temozolomide. (C) Representative images (upper panel) and survival fraction (lower panel) from colony formation assay carried out in parental and *ARH3* KO PEO1 cells treated with or without PARGi in combination with DMSO or temozolomide. (D) Representative images (upper panel) and survival fraction (lower panel) from colony formation assay carried out in parental and *ARH3* KO U2OS cells treated with or without PARGi in combination with DMSO or hydroxyurea (HU). Each experiment shown in this figure was conducted in biological and technical triplicates.

We next examined the cellular effects of treating *ARH3* KO cells with temozolomide (TMZ), a methylating agent used clinically to treat glioblastoma and still under study in other solid tumors, including ovarian cancers [58,59]. PARGi treatment of control and *ARH3* KO U2OS (Fig. 4A and Fig. S4A), COV362 (Fig. 4B and Fig. S4B), and PEO1 cells (Fig. 4C and Fig. S4C) sensitized cells to TMZ, consistent with prior findings [[60], [61], [62], [63]]. Notably, after treatment with PARGi, all *ARH3* KO cell lines showed even higher sensitivity to TMZ than their parental counterparts. These findings further indicate that blocking ADP-ribosylation removal is synthetically lethal with DNA alkylation.

Because DNA alkylation ultimately causes replicative stress [55], we asked whether loss of de-PARylation sensitizes to alkylating drugs by interfering with BER or with the replication stress response. To address this question, we challenged control and *ARH3* KO U2OS cells with hydroxyurea (HU), which generates replication stress by depleting the pool of cellular nucleotides [64,65]. We observed a similar increase in cell death in control U2OS cells and *ARH3* KO U2OS treated with the HU/PARGi combination (Fig. 4D and Fig. S4D) rather than the marked hypersensitization seen with the MMS/PARGi combination in *ARH3* KO cells (Fig. 3D). These results suggest that the synthetic lethality induced by the combination of PARGi and MMS/TMZ in *ARH3* KO cells is likely not due to impairment of the replication stress response but rather interference with the repair of methylated bases.

### Dual ARH3 and PARG enzymatic activity loss correlates with decreased PARP1/2 protein levels

We previously found that the levels of PARP1 protein, but not PARP1 mRNA, were significantly reduced in *ARH3* KO cells treated with PARGi compared to control cells [19]. Here, we assessed whether this might contribute to the increased alkylating agent sensitivity observed in *ARH3* KO cells treated with PARGi.

**Fig. 5:**
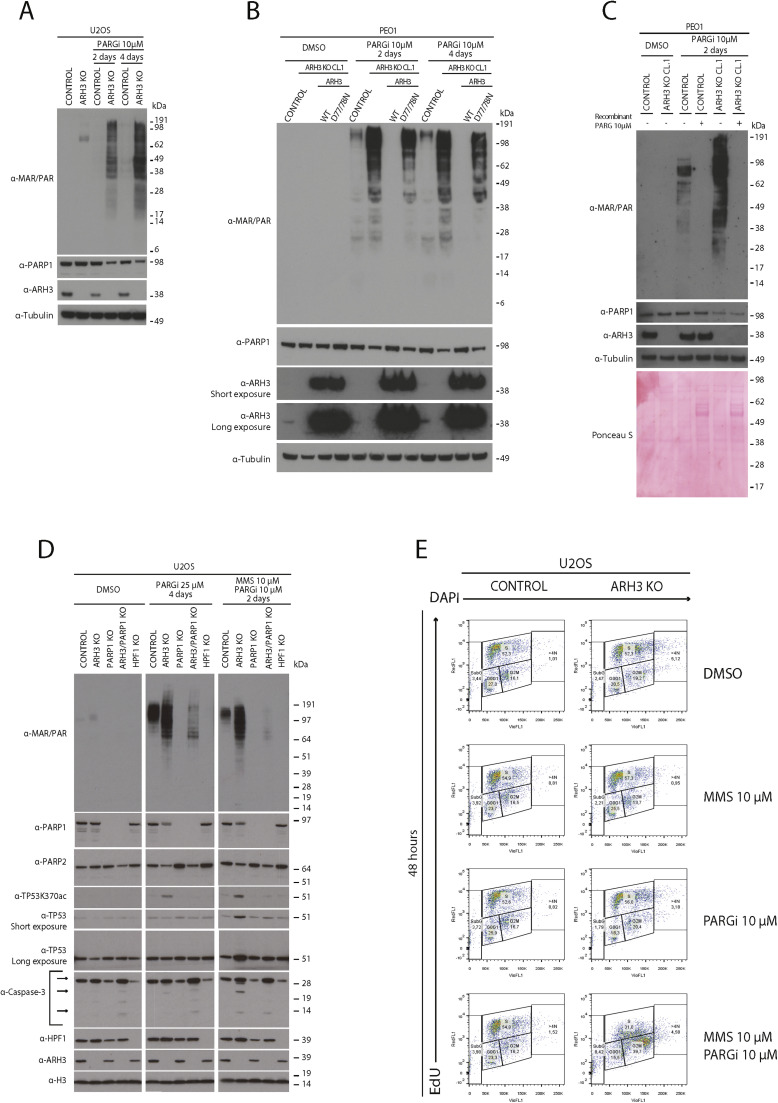
Dual ARH3 and PARG enzymatic activity loss correlates with decreased PARP1/2 protein levels. (A) Representative western blot of total cell lysates extracted from parental and *ARH3* KO U2OS cells treated with DMSO or 10 µM PARGi for two or four days. α-tubulin served as loading controls. (B) Representative western blot of total cell lysates extracted from parental PEO1, *ARH3* KO PEO1, and *ARH3* KO PEO1 complemented with wild-type ARH3 (ARH3 WT) or ARH3 D77/78N double mutant (D77/78N) treated with DMSO or 10 µM PARGi for two or four days. α-tubulin served as loading controls. (C) Representative western blotting analysis of total cell lysates extracted from control and *ARH3* KO PEO1. The cells were treated with DMSO or 10 μM PARGi for two days. The cells were lysed, and total cell extracts were incubated with or without 10 μM recombinant PARG enzyme for 30 minutes at 30°C. The cell lysates were then analyzed by western blotting using the indicated antibodies. α-tubulin and Ponceau S served as loading controls. (D) Representative western blot of total cell lysates extracted from parental and *ARH3* KO, *PARP1* KO, *ARH3/PARP1* double KO, and *HPF1* KO U2OS cells treated with DMSO or 25 µM PARGi for four days or with the combination of MMS and 10 µM PARGi for two days. α-H3 served as loading controls. (E) Representative scatterplots of flow cytometry analysis for cells were treated with DMSO, MMS, PARGi or a combination of MMS and PARGi for 48 hours and stained with EdU-DAPI. Experiments in this figure were performed in biological triplicates.

In initial experiments, immunoblotting demonstrated decreased levels of PARP1 protein in PARGi-treated *ARH3* KO U2OS cells (Fig. 5A) and PARGi-treated *ARH3* KO PEO1 cells (Fig. 5B) relative to controls, suggesting that this is a general mechanism occurring in different cell lines. Moreover, transfection with wild-type ARH3, but not the ARH3 catalytic mutant D77/78N, restored PARP1 levels (Fig. 5B), indicating that PARP1 protein levels are directly linked with functional ARH3 enzymatic activity in the presence of PARGi. To assess whether the decrease in PARP1 protein level in *ARH3* KO cells treated with PARGi was real and not because excessive PARP1 auto-PARylation obscured protein detection with the PARP1 antibody, we conducted a biochemical reaction *in vitro*. We treated the control and *ARH3* KO samples after lysis with recombinant PARG (see Fig. 5C). The enzymatic treatment did not restore PARP1 protein to the control levels, indicating that the decrease in PARP1 protein was not due to a technical artifact. Further experiments would be required to establish whether auto-PARylation in these conditions modulates PARP1 levels through the effect on protein stability, protein turnover, or by some additional mechanism.

To investigate levels of PARP1 and other proteins after altering the main components of PARP1/2 signaling by gene knockout, we treated control and *ARH3* KO, *PARP1* KO, *ARH3/PARP1* double KO, and *HPF1* KO U2OS cells with DMSO, high doses of PARGi for four days, or a combination of PARGi and MMS. Immunoblotting demonstrated a reduction in PARP1 protein levels only in *ARH3* KO cells treated with PARGi or the PARGi/MMS combination (Fig. 5D). Treatment of *ARH3* KO U2OS cells with PARGi or PARGi/MMS also reduced PARP2 protein levels. This PARP1/2 reduction, like PARP inhibition (Fig. 2C), may render cells more vulnerable to alkylating agents.

We also measured cell cycle checkpoint protein TP53 and apoptotic marker caspase-3 levels in TP53-proficient U2OS cells. Treatment of *ARH3* KO U2OS cells with high doses of PARGi for a prolonged time resulted in a modest increase in acetylation of TP53 lysine 370 (TP53K370ac) [66] but did not change total TP53 levels (Fig. 5D). In contrast, adding MMS to PARGi resulted in the strong induction of TP53K370ac, which accumulated in the nuclear fraction (Fig. S5A and S5B), and an overall increase of TP53 protein (Fig. 5D). Furthermore, we only observed caspase 3 cleavage in *ARH3* KO U2OS cells treated with PARGi and MMS (Fig. 5D), consistent with Annexin V binding results reported in Fig. 3H. Notably, neither TP53 stabilization nor caspase 3 cleavage occurred in *PARP1* KO or *ARH3/PARP1* double KO cells treated with PARGi and MMS, again indicating that PARP1 is required for the cellular toxicity of excessive PARylation induced by *ARH3* KO and PARGi in the presence of MMS.

Next, we investigated the effects of *ARH3* gene loss on proliferation and cell cycle distribution after treatment with PARGi and MMS (Fig. 5E and Fig. S5C). DNA synthesis (assessed by EdU incorporation) and cell cycle distribution (assessed by DAPI staining) did not change significantly in *ARH3* KO U2OS cells treated for 2 days with MMS compared to DMSO. PARG inhibition resulted in a slight change in the cell cycle distribution, with marginally more PARGi-treated *ARH3* KO U2OS cells in S-phase and G2-phase than the parental cells, consistent with the role of PARylation by PARP1/2 in normal DNA replication [67,68] and in preventing fork restart by suppressing RECQ1 helicase [9,38,69,70].

Compared to monotherapy treatments, the combination of MMS and PARGi resulted in more significant changes in the cell cycle distribution of *ARH3* KO U2OS cells compared to parental cells, with 37.8% ± 2.68% of *ARH3* KO cells vs. 16.8% ±0.84 % of parental U2OS cells arrested in G2 (Fig. 5E and Fig. S5C). These changes are consistent with the activation of the S/G2 checkpoint, and TP53-mediated cell cycle arrest suggested in *ARH3* KO U2OS cells by immunoblotting (Fig. 5D).

Our data collectively support the idea that a simultaneous deficiency in ARH3 and PARG is linked to reduced levels of PARP1/2 proteins, which may worsen MMS toxicity, leading to apoptotic cell death.

### Combined ARH3 knockout and PARG inhibition results in excessive alkylating agent-induced PARylation and DNA damage in response

**Fig. 6:**
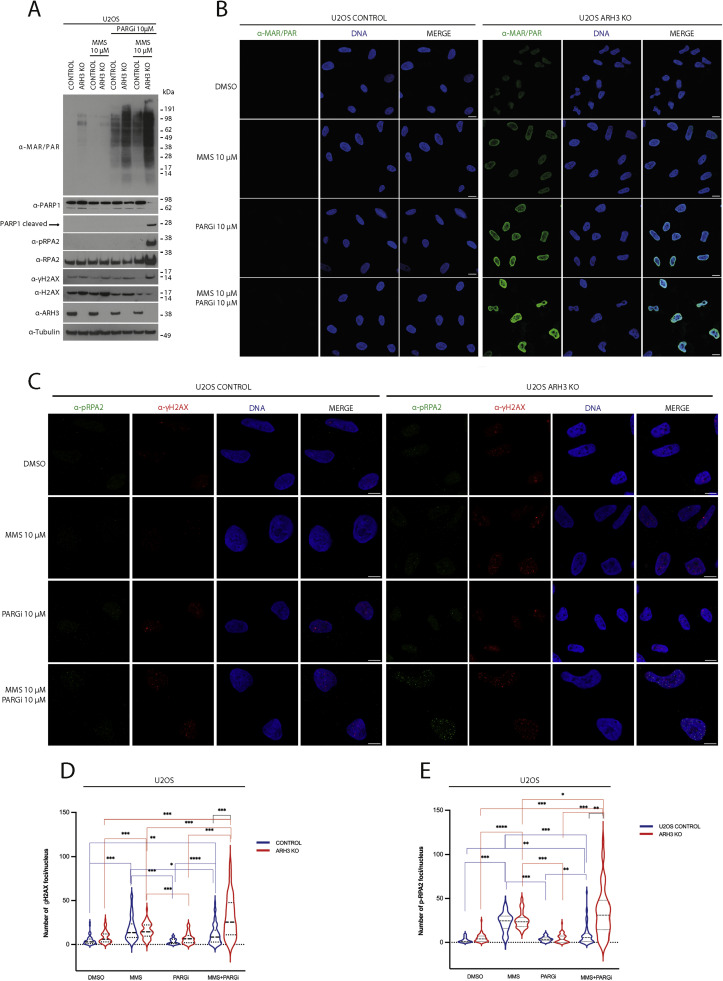
ARH3 KO combined with PARGi results in excessive PARylation and DNA damage in response to treatment with a DNA alkylating agent. (A) Total cell lysates extracted from control and *ARH3* KO U2OS cells after treatment with DMSO, MMS, PARGi or a combination of MMS and PARGi for 48 hours were then analyzed by western blotting using the indicated antibodies. α-tubulin and Ponceau S served as loading controls (B) Confocal images of control and *ARH3* KO U2OS cells treated with DMSO, MMS, PARGi or a combination of MMS and PARGi for 48 hours before detergent pre-extraction, fixation, further permeabilization and immunostaining with anti-MAR/PAR antibody (green) and DAPI (blue): Scale bars, 10 μm. Experiments were performed in biological triplicates. (C) Confocal images of control and *ARH3* KO U2OS cells treated with DMSO, MMS, PARGi, or a combination of MMS and PARGi for 48 hours before detergent pre-extraction, fixation, further permeabilization and immunostaining with the pRPA2 (green) and α-γH2AX (red) antibodies and DAPI dye (blue): Scale bars, 10 μm. The experiments were carried out three times using biological triplicates. (D-E) Quantification of pRPA2 and α-γH2AX foci. The frequency distribution of the population was analyzed, and the median was represented by a dashed line and the quartiles by dotted lines. Statistical significance was assessed using a 2-tailed Student's t-test (*p < 0.05, **p < 0.01, and ***p < 0.001).

To further address the molecular mechanisms underlying the enhanced sensitivity of *ARH3* KO cells to the PARGi/DNA alkylating agent combinatorial strategy, we examined cellular ADP-ribosylation (Fig. 6A and B). Immunoblotting (Fig. 6A) and immunofluorescence (Fig. 6B) demonstrated a dramatic increase in the ADP-ribosylation in *ARH3* KO U2OS cells treated with PARGi and MMS compared to parental cells treated with the same drug combination and *ARH3* KO treated with PARGi alone (Fig. 6A). These findings well correlated with the observed loss of viability (Fig. 3 B-H) and caspase-3 cleavage (Fig. 5D). Consistent with these results, *ARH3* KO U2OS cells treated with the PARGi and MMS combination exhibited caspase-mediated PARP1 cleavage (Fig. 6A).

Strikingly, MMS/PARGi-treated *ARH3* KO U2OS cells also contained phospho-Ser8-RPA2 (pRPA2) [65,71], suggesting the presence of single-stranded DNA, whereas pRPA2 was undetectable in parental U2OS cells under the same treatment conditions and in vehicle-treated, MMS alone-, and PARGi alone-treated *ARH3* KO cells (Fig. 6A). This observation aligns with cell fractionation data, which showed a significant accumulation of chromatin-bound RPA2 in *ARH3* KO U2OS cells treated with PARGi and MMS (Fig. S5B). Further, the *ARH3* KO U2OS cells displayed substantial levels of γH2AX, a marker of replication stress and DNA, after MMS/PARGi treatment (Figs. 6A and S6A). Confocal microscopy revealed an increase in the number and size of pRPA2 and γH2AX foci when *ARH3* KO U2OS cells were exposed to the PARGi/MMS combination compared to control and monotherapy treatments (Fig. 6C-E).

When control PEO1 cells, *ARH3* KO PEO1 and *ARH3* KO cells complemented with wild-type ARH3 or inactive ARH3 D77/78N were exposed to the PARGi/MMS combination and each agent separately (Fig. S6B), pRPA2 was observed in both parental and *ARH3* KO PEO1 cells treated with the PARGi/MMS combination, suggesting that the *BRCA2* deficient background of PEO1 cells may cause accumulation of single-stranded DNAs in parental cells in response to the combination. Moreover, the reintroduction of wild-type *ARH3*, but not ARH3 D77/78N, significantly lowered pRPA2 in *ARH3* KO PEO1 cells, indicating that ARH3 controls the accumulation of pRPA2 foci in PEO1 cells. As was the case for U2OS cells, γH2AX levels were higher in *ARH3* KO PEO1 cells treated with PARGi and MMS than in parental PEO1 cells under the same conditions (Fig. S6B) and could be reduced by introducing wild-type *ARH3* but not ARH3 D77/78N.

As shown in Fig. 5B, treating *ARH3* KO PEO1 cells with PARGi alone reduced the levels of PARP1 protein while reintroducing wild-type ARH3, but not the D77/78N mutant, restored PARP1 protein (Fig. S6B). Moreover, MMS/PARGi combinatorial treatment further decreased PARP1 in *ARH3* KO PEO1 cells compared to parental cells, leading to apoptotic PARP1 cleavage that could only be rescued by reintroducing wild-type ARH3, not the D77/78N mutant into *ARH3* KO PEO1 cells.

The findings indicate that functional de-PARylation is essential for maintaining cell homeostasis and responding to DNA damage. When the catalytic activities of ARH3 and PARG are simultaneously suppressed, cells cannot respond to alkylating agents, thus accumulating single-strand DNA and DNA damage, leading to cycle arrest and apoptosis.

## Discussion

PARPis have revolutionized the treatment of cancers with homologous recombination deficiencies [7,8]. Although these agents have been in clinical use since 2014, the molecular mechanisms controlled by PARP1/2 and novel systems tuning PARP signaling continue to be uncovered [10,11,14,16,17,72].

ARH3 and PARG are the two main ADP-ribosyl hydrolases responsible for reversing PARP1/2-mediated protein PARylation [57]. Importantly, loss of PARG expression has been identified as one of the PARPi resistance mechanisms [35,73], underscoring the potential of de-PARylation systems in modulating cancer cell drug responses [9,36,38,40,43]. Despite ARH3 and PARG having epistatic molecular functions, genetic *ARH3* loss sensitizes cancer cells to PARGi [19]. The combined loss of PARG and ARH3 functions may lead to cellular toxicity due to imbalances in cellular pathways related to serine-linked PARylation, for instance, resulting in histone acetylation changes, altered transcriptional programs, and telomere extension through alternative lengthening of telomeres (ALT) [19,20,74]. Furthermore, ARH3 and PARG ADP-ribosyl hydrolytic activities have been recently shown to be involved in the catalysis of other types of amino acid linkages, such as tyrosine-linked ADP-ribosylation [75]. Excessive signaling from tyrosine-linked ADP-ribosylation may also play a role in cell death through unknown mechanisms [75]. Altogether, these observations suggest that inhibiting PARG and ARH3 enzymatic activities could be a promising therapeutic strategy, especially for PARPi-resistant tumors that modulate PARG protein levels [35].

The primary function of PARP1/2-dependent ADP-ribosylation is to respond to DNA damage. However, the impact of simultaneously down-regulating the ADP-ribosyl hydrolases PARG and ARH3 on genome stability—and its potential as a source of genotoxicity—has not been explored yet. By treating PARGi-treated *ARH3* KO cells with genotoxins that target different DNA repair pathways, we observed a significant increase in the cancer cells’ vulnerability to the alkylating agents MMS and TMZ, thus supporting the role of PARP1 and PARP2 in repairing DNA damage caused by alkylating agents. Although the direct involvement of PARP1/2 in the BER process is debated, as reviewed by Chaudhuri and Nussenzweig [76], the formation of apurinic-apyrimidinic sites by AP endonucleases indirectly creates SSBs, whose repair has been extensively shown to depend on PARP1/2-dependent PARylation [4,76]. The almost complete suppression of de-PARylation achieved in PARGi-treated *ARH3* KO cells allows the visualization of PARylation in response to alkylating genotoxins. This observation suggests that, directly or indirectly, alkylating genotoxins activate the PARP1/PARP2-dependent modification of proteins. However, the hyper-PARylation of DNA repair proteins induced by the loss of ARH3 and PARG activities may interfere with the resolution of the alkylator-induced DNA damage. Consistent with this model, ARH3-deficient cells treated with PARGi and alkylating agents accumulate pRPA2 (a marker of ssDNAs) and γH2AX (a marker of DNA damage), which, in turn, triggers the G2/M cell cycle checkpoint, ultimately leading to apoptotic cell death.

This study suggests a novel strategy for targeting cancer cells: inhibiting ADP-ribosyl hydrolases to enhance the toxicity of TMZ in cancer treatment. Following a distinct rationale compared with this study, the use of PARGi in combination with TMZ was explored to treat the isocitrate dehydrogenase (IDH) mutant glioblastoma cells [61,63]. Oncogenic mutations in the IDH1 and IDH2 enzymes in cancer facilitate the biochemical reaction transforming alpha-ketoglutarate (α-KG) into the oncometabolite 2-hydroxyglutarate (2-HG)—the overproduction of 2-HG results in metabolic abnormalities, including significantly diminished basal levels of NAD^+^ [77]. Thus, NAD^+^ depletion can be further exacerbated through PARGi treatment and alkylating chemotherapy [61,63]. While we do not dismiss the role of metabolic outcomes from PARG inhibition on NAD^+^ metabolism, our findings indicate that specific cell toxicity can be achieved by stimulating BER using alkylating genotoxins. Indeed, such a cell toxicity phenotype is not observed with other chemotherapeutics that activate PARP-dependent/NAD^+^-depleting pathways, such as cisplatin, which mainly involves repair by Nucleotide Excision Repair (NER) [76,78].

When *ARH3* knockout cells were exposed to PARGi alone, we observed another phenotype: a steady decrease in PARP1/2 protein levels in all cell models we examined. This effect of hyper PARP1/2 autoPARylation is likely not due to the decreased PARP gene transcription [19], but it may be caused by increased protein turnover, decrease of protein stability, or some other mechanism. This reduction of PARP1 and PARP2 may partly contribute to increased alkylating agent sensitivity in *ARH3* KO cells, in accordance with previous reports that PARP1 loss of function sensitizes cells to these agents [56].

Collectively, the present data support the importance of the PARP1/2 and PARG/ARH3 regulatory axis in cellular homeostasis. An imbalance between the writers and erasers of ADP-ribosylation can significantly impact how cancer cells respond to PARPis and alkylating agents. In this regard, ARH3, for which specific inhibitors are not yet available, may represent a new target for cancer therapy. When ARH3 inhibitors become available, combining them with alkylating agents will likely be effective, particularly in tumors where PARG downregulation contributes to PARPi resistance. Our results also encourage research into relationships between other ADP-ribosyl hydrolases that act redundantly against PARP1-dependent modification, e.g., the recently discovered interplay between PARG and TARG1 [21,72,79].

## Consent for publication

All authors approved the final manuscript and submission to this journal.

## Availability of Data and Material

All research reagents generated by the authors will be made available on request from the Lead contact.

## Funding

This work was supported by the 10.13039/100014008Ovarian Cancer Research Alliance (813369) to LP, IA, SJW, SHK; PRIN 2022 (2022R85H27_LS3) to LP; AIRC (IG grant 23218) to RMM; 10.13039/100010269Wellcome Trust (210634, 223107, 302632) to IA; 10.13039/501100008886Cancer Research United Kingdom (C35050/A22284) to IA; 10.13039/100000871Mayo Clinic SPORE in Ovarian Cancer (P50 CA136393) to SHK.

## Lead contact

Further information and requests for resources and reagents should be directed to and will be fulfilled by the Lead Contact, Luca Palazzo (luca.palazzo@unina.it)***.***

Key resources table

**Table:** 

REAGENT or RESOURCE	SOURCE	IDENTIFIER
Antibodies		
anti-poly/mono ADPr (rabbit monoclonal)	Cell Signaling	Cat# 83732 AB_2749858
anti-H2AX (rabbit polyclonal)	Cell Signaling	Cat# 2595 AB_10694556
anti-PARP1 (rabbit monoclonal)	Abcam	Cat# ab32138 AB_777101
anti-PARP1 (mouse monoclonal)	BD Biosciences	Cat# 556494 AB_396433
anti-γH2AX (mouse monoclonal)	Cell Signaling	Cat# D7T2V
anti-α-tubulin (mouse monoclonal)	Sigma-Aldrich	Cat# T607 AB_477582
anti-β-tubulin (rabbit polyclonal)	Abcam	Cat# ab6046 AB_2210370
anti-RPA32 p-S4/8 (rabbit polyclonal)	Bethyl	Cat# A300-245A AB_210547
anti-Phospho-RPA32/RPA2 (Ser8) (rabbit polyclonal)	Cell Signaling	Cat# 54762 AB_2799471
anti-RPA32 (rabbit polyclonal)	Cell Signaling	Cat# 52448 AB_2750889
anti-PAN-ADP-RIBOSE binding reagent	Merck Millipore	Cat# MABE1016 AB_2665466
anti-Poly(ADP-ribose) (rabbit polyclonal)	Enzo Life Sciences	Cat# ALX-210-890A-0100
anti-ADPRHL2 (rabbit monoclonal)	Sigma-Aldrich	Cat# HPA027104 AB_10601330
anti-histone H3 (rabbit polyclonal)	Millipore	Cat# 07–690 AB_417398
anti-p53 (mouse monoclonal)	Santa Cruz	Cat# sc-126 AB_628082
anti-p53 K370ac	Cell Signaling	
anti-p53 K382ac	Cell Signaling	Cat# 2525 AB_330083
anti-Caspase 3 (rabbit monoclonal)	Cell Signaling	Cat# 14220 AB_2798429
anti-Caspase 7 (rabbit monoclonal)	Cell Signaling	Cat# 12827 AB_2687912
anti-HPF1/C4orf27 (rabbit polyclonal)	NovusBio	Cat# NBP1-93973
anti-H2A (rabbit polyclonal)	Abcam	Cat# ab18255 AB_470265
anti-PARP2 (mouse monoclonal)	Millipore	Cat# MABE18 AB_11214439
anti-lamin A (rabbit polyclonal)	Abcam	Cat# ab26300 AB_775965
Goat polyclonal anti-mouse, HRP-conjugated	Agilent	Cat# P0447 AB_2617137
Swine polyclonal anti-rabbit, HRP-conjugated	Agilent	Cat# P0399 AB_2617141
Alexa Fluor 488 anti-rabbit IgG	Life Technologies	Cat# A32731
Alexa Fluor 594 anti-mouse IgG	Life Technologies	Cat# A32744
Chemicals, peptides, and recombinant proteins		
PDD00017273 (PARGi)	MCE	Cat# HY-108360
Olaparib	MCE	Cat# HY-10162
Methyl methanesulfonate (MMS)	Merck Millipore	Cat# 129925
Crystal violet	Merck Millipore	Cat# C0775
PhosSTOP	Merck Millipore	Cat# 4906837001
cOmplete™, EDTA-free Protease Inhibitor Cocktail	Merck Millipore	Cat# 11873580001
Benzonase	Merck Millipore	Cat# E1014
Puromycin	Invivogen	ANT-PR-1
Blasticidin	Invivogen	ANT-PR-1
NuPAGE LDS sample buffer	Life Technologies	Cat# NP0008
NuPAGE Novex 4–12% Bis-Tris gel	Invitrogen	Cat# WG1402A
5-Fluorouracil (5-FU)	Sigma	Cat# F6627
Camptothecin (CPT)	Selleckchem	Cat# S1288
Cisplatin	Merck Millipore	Cat# P4394
Paclitaxel	Abcam	Cat# ab120143
Hydroxyurea	Merck Millipore	Cat# 1016970001
Temozolomide (TMZ)	MCE	Cat# HY-17364
DAPI	Merck Millipore	Cat# D9542
TCEP	Merck Millipore	Cat#646547
Trichostatin A	Merck Millipore	Cat# T8552
Recombinant human PARG protein	Fontana et al., 2017	N/A
Critical commercial assays		
TransIT-LT1 Transfection Reagent	Mirus Bio	Cat# MIR 2300
CellTiter 96® AQueous One Solution Cell Proliferation Assay (MTS)	Promega	Cat# PRO-G3580
Click-iT Plus EdU Alexa Fluor 647 Flow Cytometry Assay Kit	Life Technologies	Cat# C10419
Subcellular Protein Fractionation kit for Cultured Cells	Thermo Fisher Scientific	Cat#78840
FuGENE	Promega	Cat# E2311
LR Clonase II enzyme mix	Invitrogen	Cat# 11791020
Click-iT Plus EdU Alexa Fluor 647 Flow Cytometry Assay Kit	Life Technologies	Cat# C10419
FITC Annexin V/Dead Cell Apoptosis Kit	Invitrogen	Cat# V13242
Experimental models: Cell lines		
Human: U2OS cells	ATCC	HTB-96 CVCL_0042
Human: U2OS ARH3 KO cells	Fontana et al., 2017	N/A
Human: U2OS ARH3 KO cells complemented with untagged ARH3 WT	Prokhorova et al., 2021	N/A
Human: U2OS ARH3 KO cells complemented with untagged ARH3 D77/78N	Prokhorova et al., 2021	N/A
Human: PEO1 cells	Fergus Couch, Mayo Clinic	
Human: PEO1 ARH3 KO cells	This paper	
Human: PEO1 ARH3 KO cells complemented with untagged ARH3 WT	This paper	N/A
Human: PEO1 ARH3 KO cells complemented with untagged ARH3 D77/78N	This paper	N/A
Human: COV362 cells	Hurley, R.M. et al., 2019	
Human: COV362 ARH3 KO cells	This paper	N/A
Human: OVCAR8 cells	Hurley, R.M. et al., 2019	
Human: OVCAR8 ARH3 KO cells	This paper	N/A
Oligonucleotides		
sgRNA #210 (GCGCTGCTCGGGGACTGCGT)	Fontana et al., 2017	
sgRNA #212 (GGGCGAGACGTCTATAAGGC)	Fontana et al., 2017	
sgRNA #1(CCACCTCAACGTCAGGGTG)	This paper	
sgRNA #2 (TGGGTTCTCTGAGCTTCGT)	This paper	
sgRNA #1(CAGCAGAATTCCCCGATCCG)	This paper	
sgRNA #2 (TCGGCGGTGGCGGGAAGCGC)	This paper	
Recombinant DNA		
pX459(1.1) (plasmid)	Addgene	Cat# 108292
pLX304 (plasmid)	Addgene	Cat# 25890
pCMV-VSV-G (plasmid)	Addgene	Cat# 8485
pCMV-dR8.2 (plasmid)	Addgene	Cat #8455
Software and algorithms		
ImageJ	NIH	
Prism 10	GraphPad	
ICY	INSTITUT PASTEUR	
Zen Microscopy software	ZEISS	
FlowJo software	BD Biosciences	

## CRediT authorship contribution statement

**Rocco Caggiano:** Investigation. **Evgeniia Prokhorova:** Investigation. **Lena Duma:** Investigation. **Kira Schützenhofer:** Investigation. **Raffaella Lauro:** Investigation. **Giuliana Catara:** Investigation. **Rosa Marina Melillo:** Writing – review & editing, Conceptualization. **Angela Celetti:** Writing – review & editing, Conceptualization. **Rebecca Smith:** Investigation. **S John Weroha:** Writing – review & editing, Supervision, Project administration, Funding acquisition, Formal analysis, Conceptualization. **Scott H Kaufmann:** Writing – review & editing, Supervision, Project administration, Funding acquisition, Formal analysis, Conceptualization. **Ivan Ahel:** Writing – review & editing, Supervision, Project administration, Funding acquisition, Formal analysis, Conceptualization. **Luca Palazzo:** Writing – review & editing, Writing – original draft, Supervision, Project administration, Investigation, Funding acquisition, Formal analysis, Conceptualization.

## Declaration of competing interest

The authors declare that they have no known competing financial interests or personal relationships that could have appeared to influence the work reported in this paper.

## Appendix Supplementary materials

Image, application 1

Image, application 2

Image, application 3

## Footnotes

Supplementary material associated with this article can be found, in the online version, at doi:10.1016/j.neo.2024.101092.
