# Supplementary figures and images for: Suppression of ADP-ribosylation reversal triggers cell vulnerability to alkylating agents
Source: Neoplasia. 2024 Nov 29;59:101092. doi: 10.1016/j.neo.2024.101092 (PMC11648251; doi:10.1016/j.neo.2024.101092)

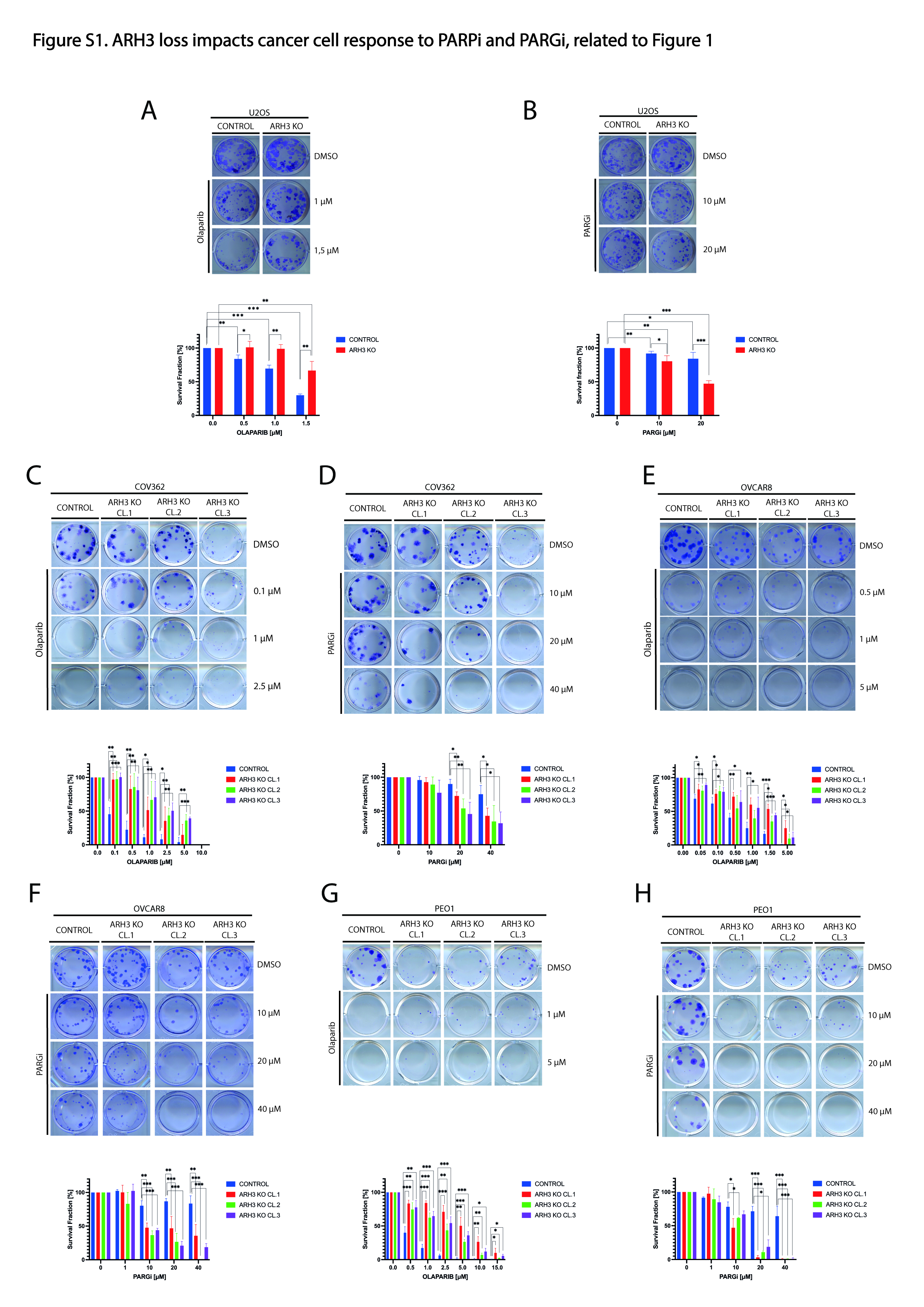

Supplement: Supplementary file 1 [file mmc1.zip › S1.tif]

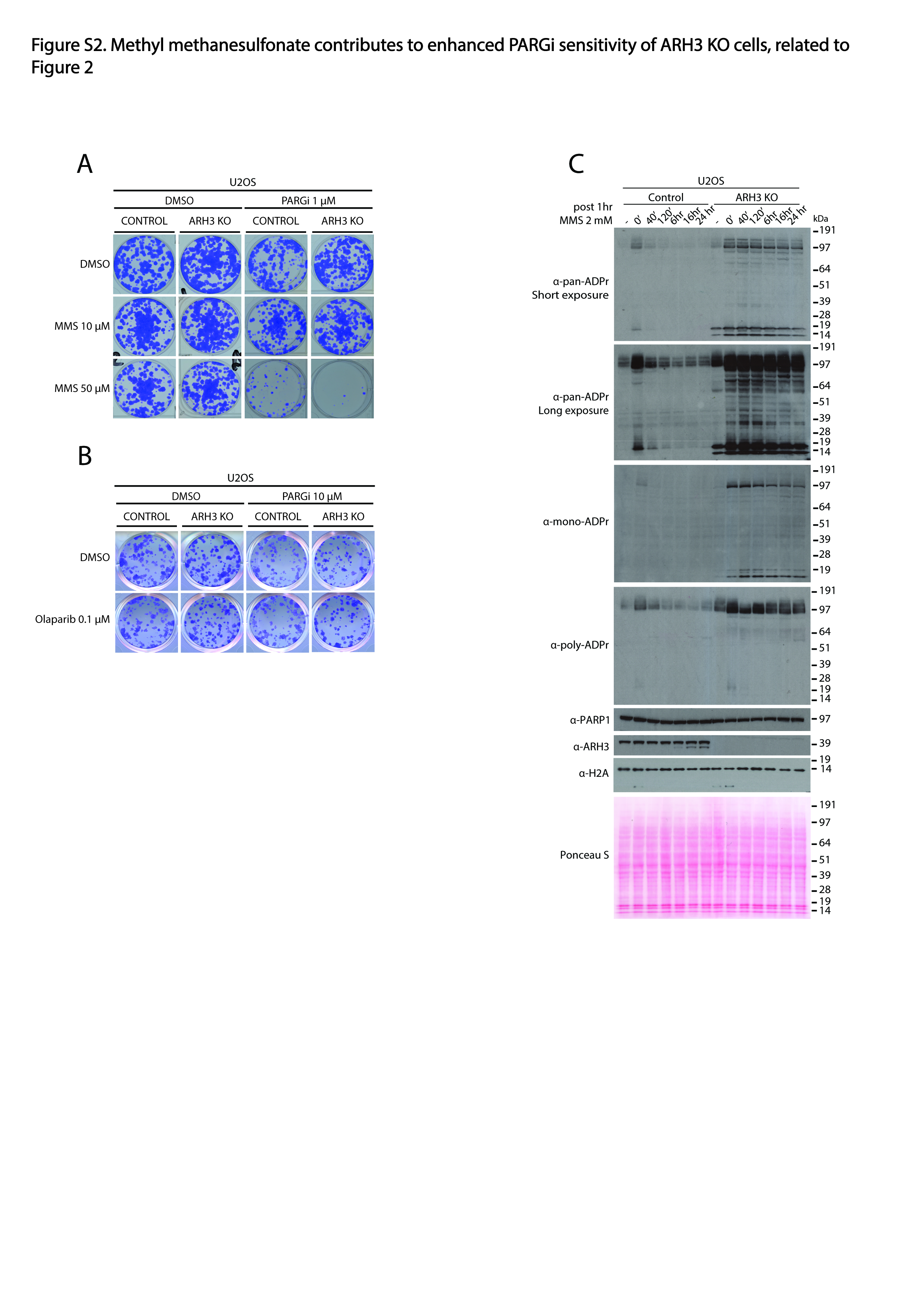

Supplement: Supplementary file 1 [file mmc1.zip › S2.tif]

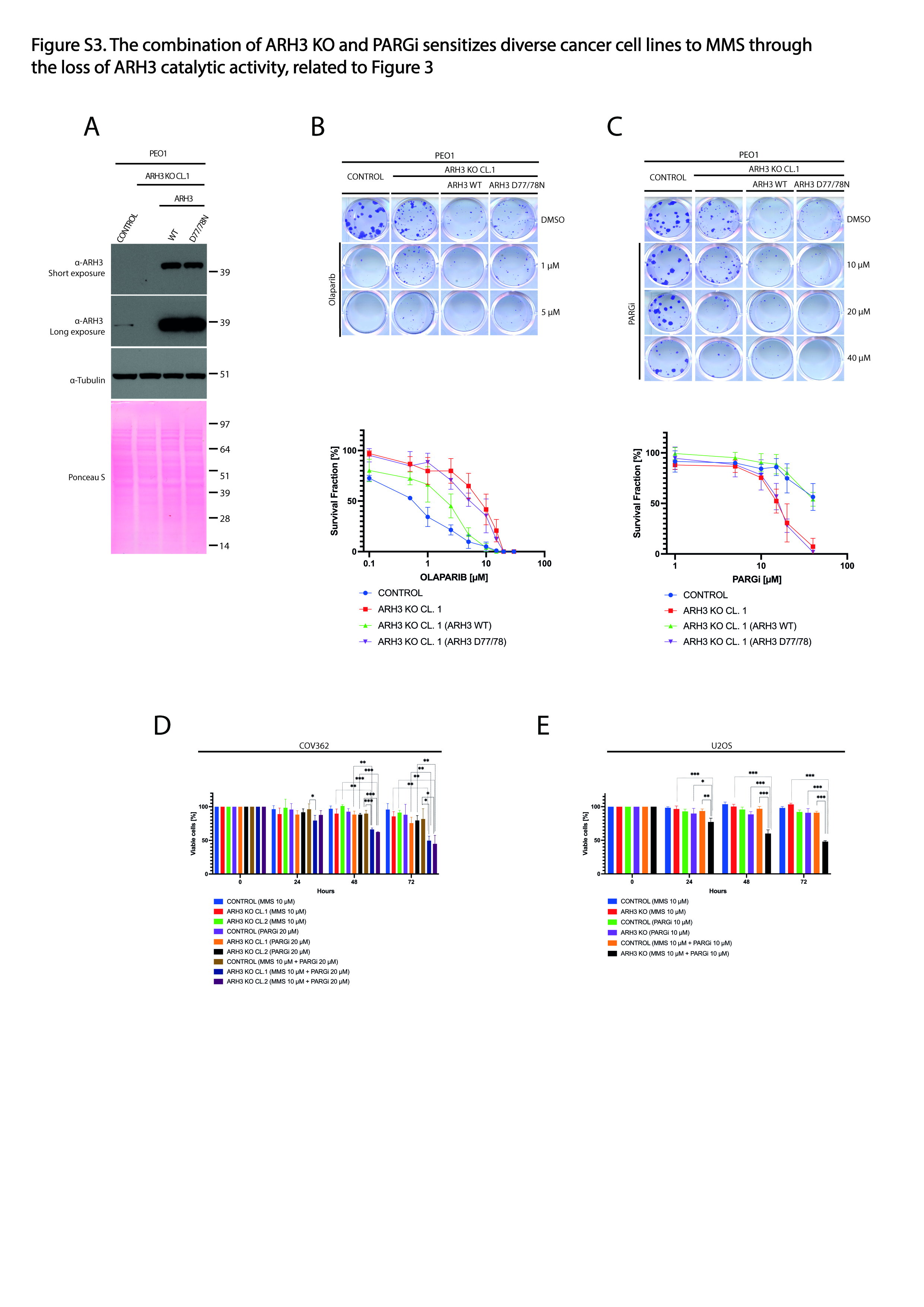

Supplement: Supplementary file 1 [file mmc1.zip › S3.tif]

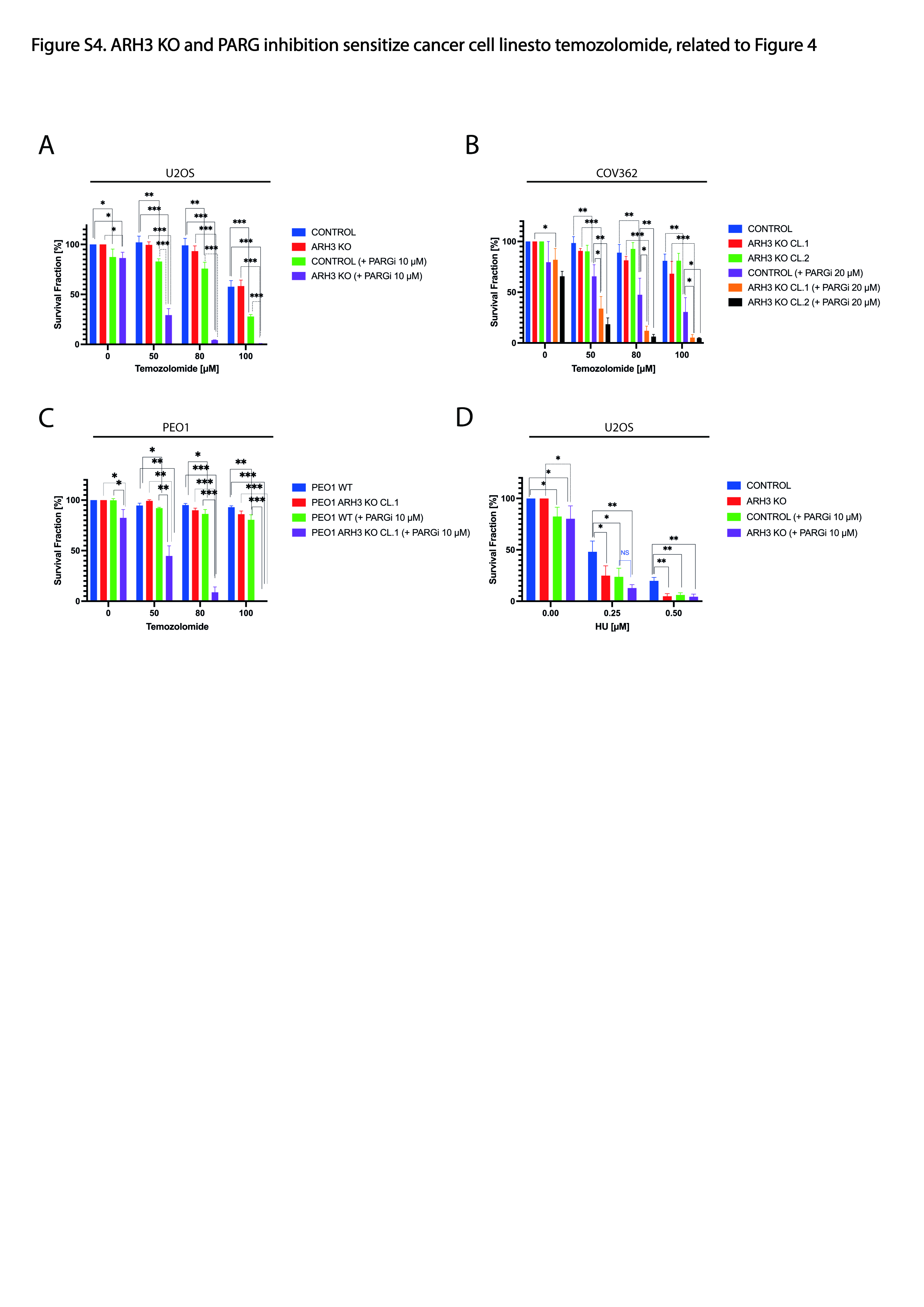

Supplement: Supplementary file 1 [file mmc1.zip › S4.tif]

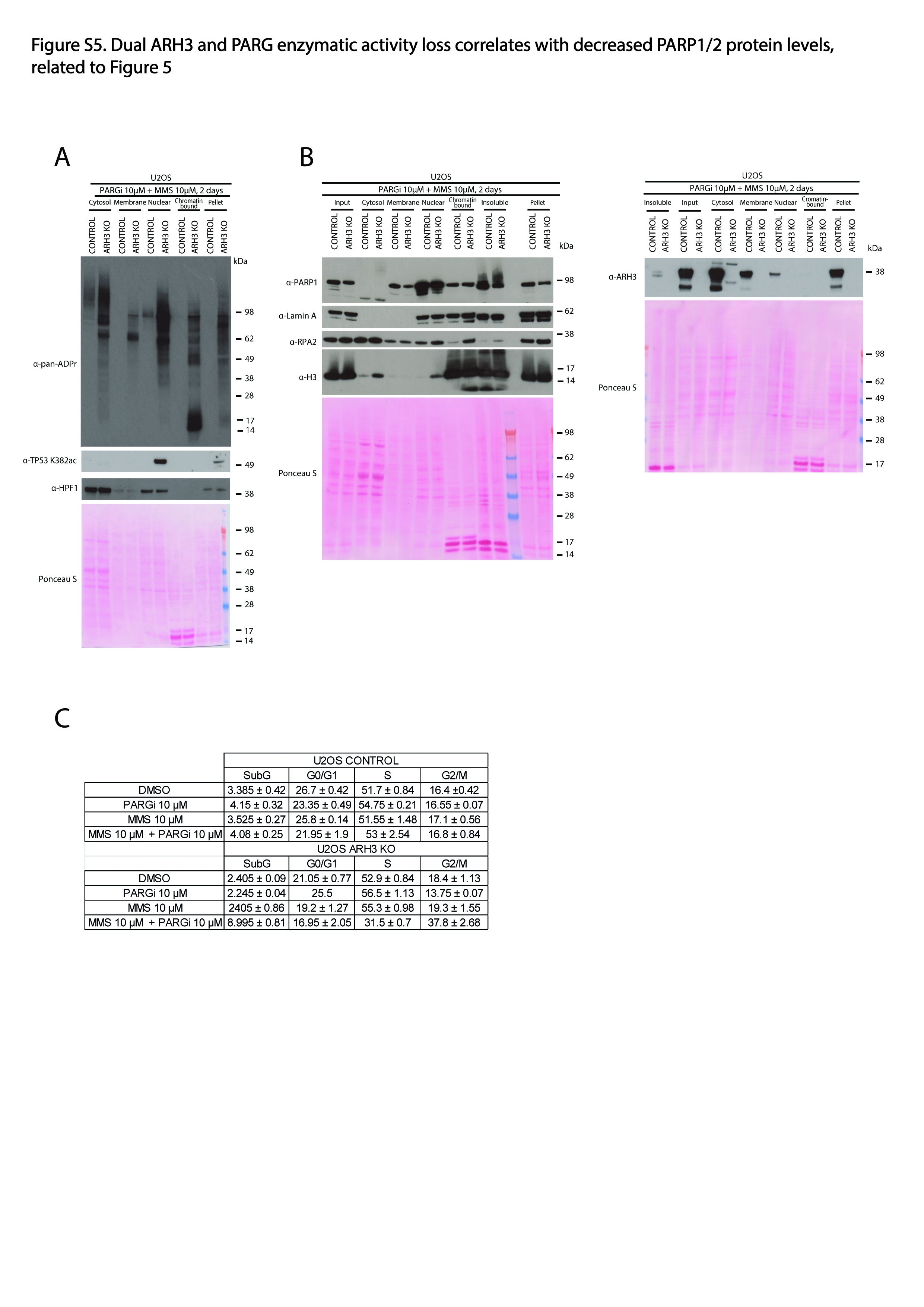

Supplement: Supplementary file 1 [file mmc1.zip › S5.tif]

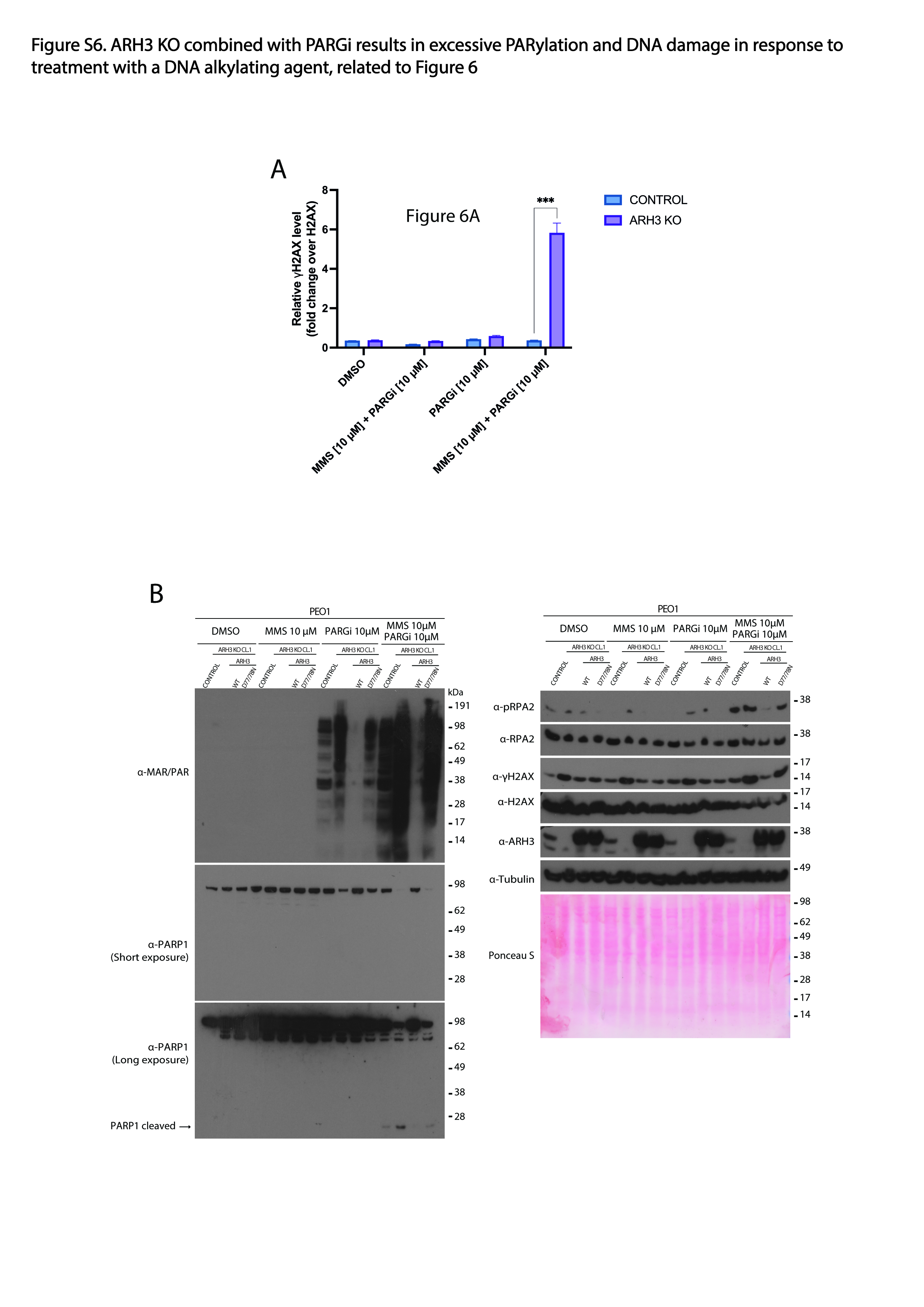

Supplement: Supplementary file 1 [file mmc1.zip › S6.tif]
