# Supplementary material for: Suppression of ADP-ribosylation reversal triggers cell vulnerability to alkylating agents
Source: Neoplasia. 2024 Nov 29;59:101092. doi: 10.1016/j.neo.2024.101092 (PMC11648251; doi:10.1016/j.neo.2024.101092)
Supplement: Supplementary file 2 [file mmc2.docx]

**SUPPLEMENTAL INFORMATION**

**FOR**

**Suppression of ADP-ribosylation reversal triggers cell vulnerability to alkylating agents.**

Rocco Caggiano, Evgeniia Prokhorova, Lena Duma, Kira Schützenhofer,

Raffaella Lauro, Giuliana Catara, Rosa Marina Melillo, Angela Celetti, Rebecca Smith,

S John Weroha, Scott H Kaufmann, Ivan Ahel, Luca Palazzo

***Supplemental Figure Legends***

**Figure S1. ARH3 loss impacts cancer cell response to PARPi and PARGi.** (A) Representative images (upper panel) and relative quantification (lower panel) of colony formation assays plotted in the left panel of Figure 1C. Control and *ARH3* KO U2OS cells were grown in DMSO or olaparib at the indicated concentrations. (B) Representative images (upper panel) and relative quantification (lower panel) of colony formation assays plotted in the right panel of Figure 1C. Briefly, control and *ARH3* KO U2OS cells were grown in the presence of DMSO or PARGi at the indicated concentrations. (C) Representative images (upper panel) and relative quantification (lower panel) of colony formation assays plotted in the left panel of Figure 1D. Control and independent clones of *ARH3* KO COV362 cells were grown in the presence of DMSO or olaparib at the indicated concentrations. (D) Representative images (upper panel) and relative quantification (lower panel) of colony formation assays plotted in the right panel of Figure 1D. Briefly, control and independent clones of *ARH3* KO COV362 cells were grown in the presence of DMSO or PARGi at the indicated concentrations. (E) Representative images (upper panel) and relative quantification (lower panel) of colony formation assays plotted in the left panel of Figure 1E. Control and independent clones of *ARH3* KO OVCAR8 cells were grown in the presence of DMSO or olaparib at the indicated concentrations. (F) Representative images (upper panel) and relative quantification (lower panel) of colony formation assays plotted in the right panel of Figure 1E. Control and independent clones of *ARH3* KO OVCAR8 cells were grown in the presence of DMSO or PARGi at the indicated concentrations. (G) Representative images (upper panel) and relative quantification (lower panel) of colony formation assays plotted in the left panel of Figure 1F. Control and independent clones of *ARH3* KO COV362 cells were grown in the presence of DMSO or olaparib at the indicated concentrations. (H) Representative images (upper panel) and relative quantification (lower panel) of colony formation assays plotted in the right panel of Figure 1F. Control and independent clones of *ARH3* KO COV362 cells were grown in the presence of DMSO or PARGi, which was used at the indicated concentrations. Each experiment was performed in biological and technical triplicates. Quantification data are shown as mean ± SD. Statistical significance was evaluated by using a 2-tailed Student's t-test (∗p < 0.05, ∗∗p < 0.01, and ∗∗∗p < 0.001).

**Figure S2. Methyl methanesulfonate contributes to enhanced PARGi sensitivity of ARH3 KO cells.** (A) Representative images of colony formation assay in control and *ARH3* KO U2OS cells treated with DMSO or PARGi at the indicated concentrations. Cells were grown as above with or without MMS at the indicated concentrations. The experiment was performed in biological and technical triplicates. (B) Representative western blotting analysis of total cell lysates extracted from control and *ARH3* KO U2OS cells. The cells were treated with DMSO or 2 mM MMS for 1 hour. After the MMS washout, the cells were harvested at the indicated time points. The total cell lysates were then analyzed by western blotting using the indicated antibodies. α-H2A and Ponceau S served as loading controls. (C) Representative images of colony formation assay in control and *ARH3* KO U2OS cells treated with DMSO or PARGi at the indicated concentrations. Cells were grown as above with or without olaparib at the indicated concentration. The experiment was performed in biological and technical triplicates.

**Figure S3. The combination of ARH3 KO and PARGi sensitizes diverse cancer cell lines to MMS through the loss of ARH3 catalytic activity.** (A) Representative western blotting analysis of ARH3 protein levels in control and *ARH3* KO PEO1 cells, as well in *ARH3* KO PEO1 cells that were complemented either with wild-type ARH3 (ARH3 WT) or catalytically inactive ARH3 mutant (ARH3 D77/78N) through lentiviral infection. α-Tubulin and Ponceau S served as loading controls. (B) Representative images (upper panel) and relative quantification with statistics (lower panel) of colony formation assays conducted in control and *ARH3* KO PEO1 cells. The assays were also conducted in *ARH3* KO PEO1 cells complemented with either wild-type ARH3 (ARH3 WT) or catalytically inactive ARH3 mutant (ARH3 D77/78N). The cells were treated with DMSO or olaparib at the indicated concentrations. (C) Representative images (upper panel) and relative quantification with statistics (lower panel) of colony formation assays conducted in control and *ARH3* KO PEO1 cells. The assays were also conducted in *ARH3* KO PEO1 cells complemented with either wild-type ARH3 (ARH3 WT) or catalytically inactive ARH3 mutant (ARH3 D77/78N). Cells were treated with DMSO or PARGi at the indicated concentrations. Experiments were performed in biological triplicates. (D-E) Cell viability analysis in control and *ARH3* KO COV362 (D) and U2OS (E) cells. Cells were grown at the indicated conditions for 24-48-72 hours and cell survival was measured using the CellTiter 96 AQueous One Solution (Promega). Each experiment was conducted with technical quintuplicates and biological triplicates. The quantified data are presented as mean ± SD. Statistical significance was assessed using a 2-tailed Student's t-test (with significance denoted as ∗p < 0.05, ∗∗p < 0.01, and ∗∗∗p < 0.001).

**Figure S4. ARH3 KO and PARG inhibition sensitize cancer cell lines to temozolomide.** (A) The results of the experiment shown in Figure 4A are quantified. (B) The results of the experiment shown in Figure 4B are quantified. (C) The results of the experiment shown in Figure 4C are quantified. (D) The results of the experiment shown in Figure 4D are quantified. Each experiment was performed in biological and technical triplicates. Quantification data are shown as mean ± SD. Statistical significance was evaluated by using a 2-tailed Student's t-test (∗p < 0.05, ∗∗p < 0.01, and ∗∗∗p < 0.001). Please note the following abbreviation: NS denotes "not statistically significant”.

**Figure S5. Dual ARH3 and PARG enzymatic activity loss correlates with decreased PARP1/2 protein levels.** (A-B) Representative western blotting analysis of total lysates and subcellular fractions extracted from control and *ARH3* KO U2OS cells. Cells were treated with MMS and PARGi for 48 hours. The total lysates (input) and the cell fractions were analyzed by western blotting using the indicated antibodies. α-H3 and α-Lamin A served as cell fractionation controls, Ponceau S served as a loading control. (C) Table shows the distribution of cell cycle phases (subG, G0/G1, S, G2/M) in control and ARH3 KO U2OS cells under the indicated treatment. Mean ± SD values were obtained from three independent experiments.

**Figure S6. ARH3 KO combined with PARGi results in excessive PARylation and DNA damage in response to treatment with a DNA alkylating agent.** (A) Image J quantification of gel bands on γH2AX western blots shown in Fig. 6A. Data indicate the fold-change of α-γH2AX levels compared to total α-H2AX levels. The experiment was performed in biological triplicates. Quantification data are shown as mean ± SD. Statistical significance was evaluated by using a 2-tailed Student's t-test (∗p < 0.05, ∗∗p < 0.01, and ∗∗∗p < 0.001). (B) Representative western blotting analysis of total cell lysates extracted from control and *ARH3* KO PEO1 cells, as well as from *ARH3* KO PEO1 cells complemented with either wild-type ARH3 (ARH3 WT) or catalytically inactive ARH3 mutant (ARH3 D77/78N). Cells were treated with DMSO, MMS, PARGi or a combination of MMS and PARGi for 48 hours. The cell lysates were then analyzed by western blotting using the indicated antibodies. α-Tubulin and Ponceau S served as loading controls.
