# Supplementary material for: Suppression of ADP-ribosylation reversal triggers cell vulnerability to alkylating agents
Source: Neoplasia. 2024 Nov 29;59:101092. doi: 10.1016/j.neo.2024.101092 (PMC11648251; doi:10.1016/j.neo.2024.101092)

FIGURE 1B

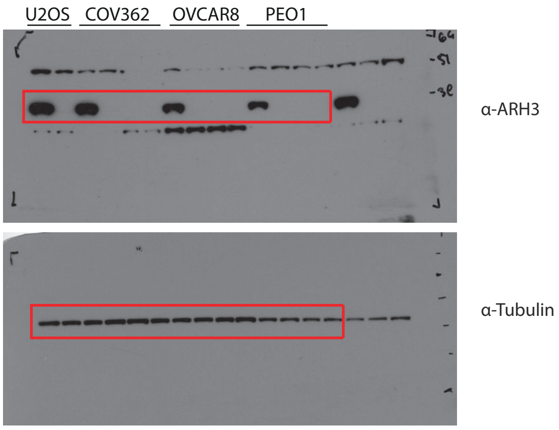

FIGURE 3A

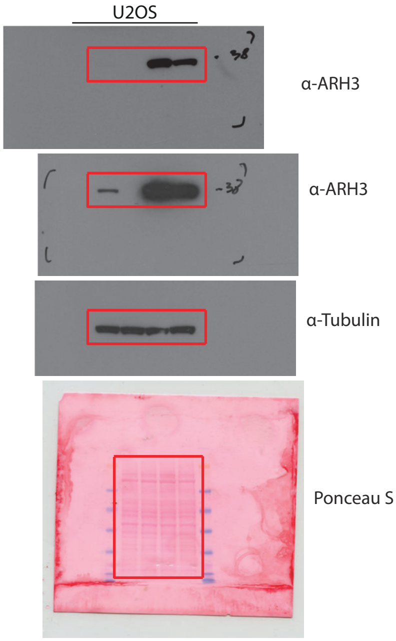

FIGURE 5A

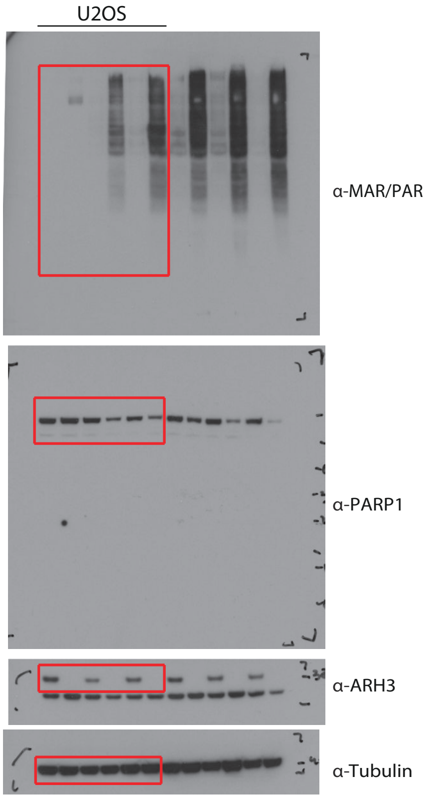

FIGURE 5B

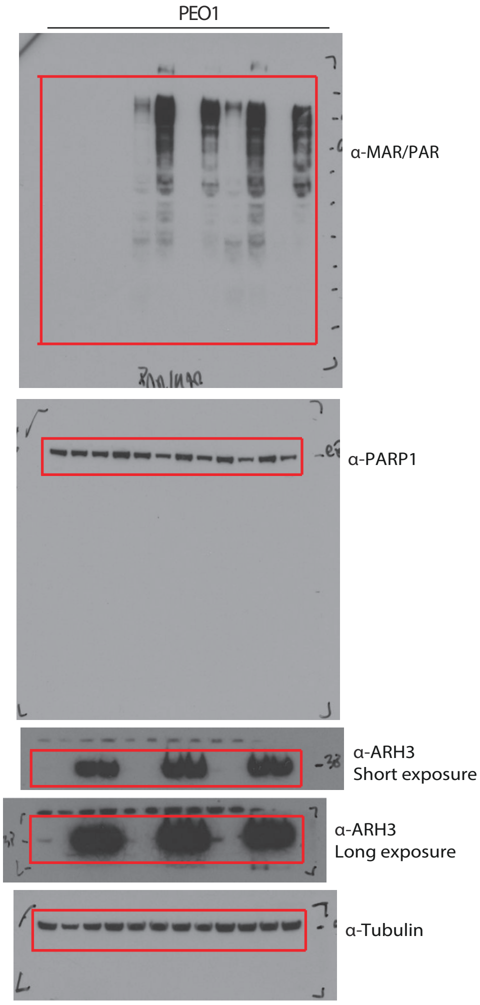

FIGURE 5C

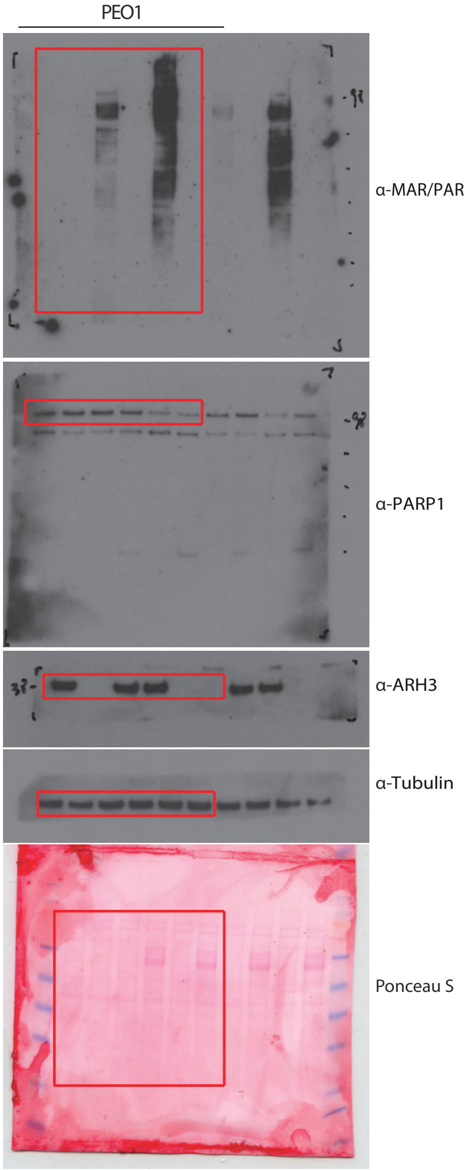

FIGURE 5D

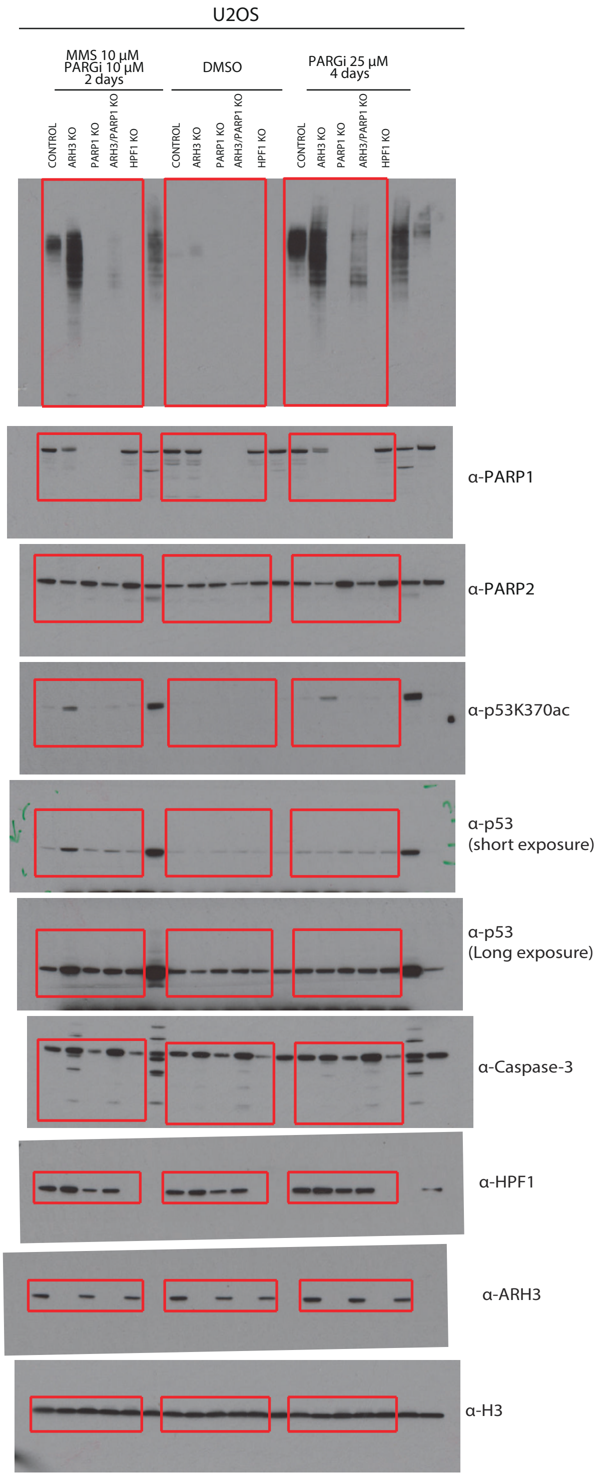

FIGURE 6A

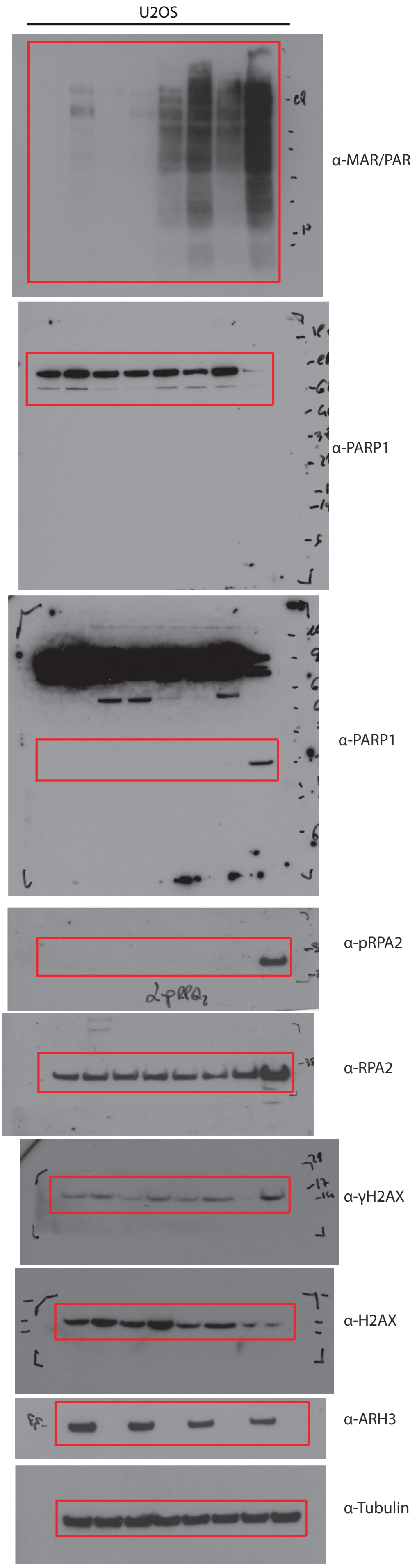

FIGURE S2B

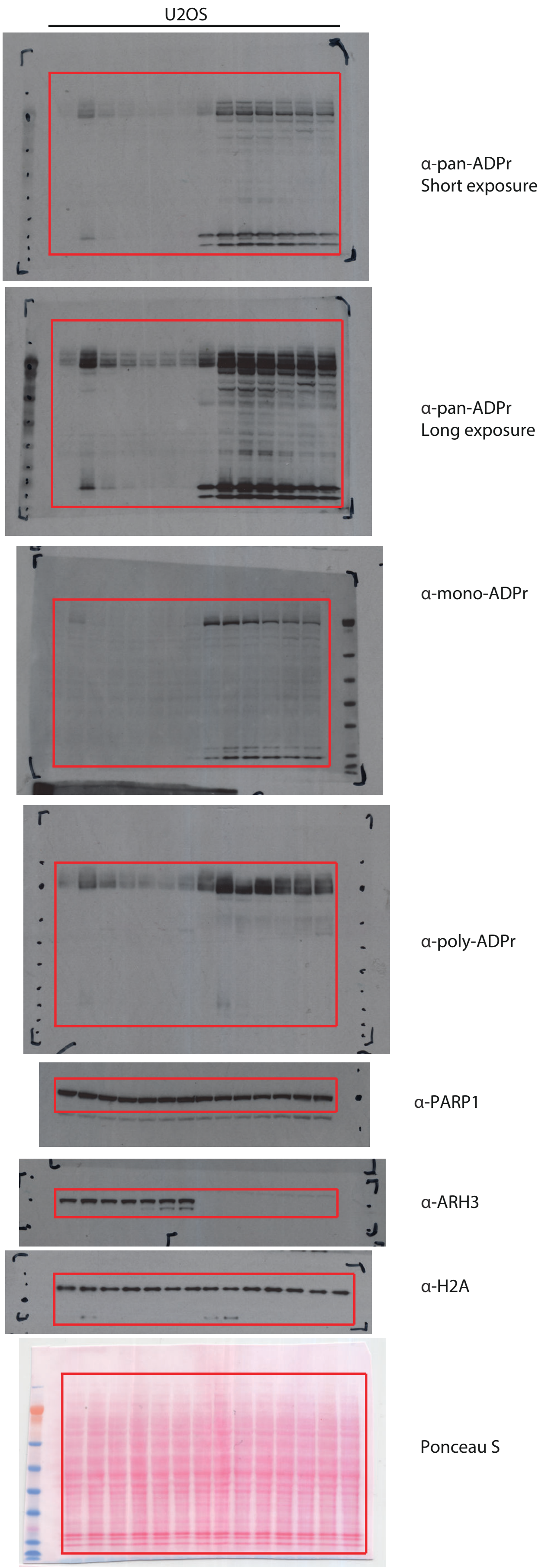

FIGURE S3A

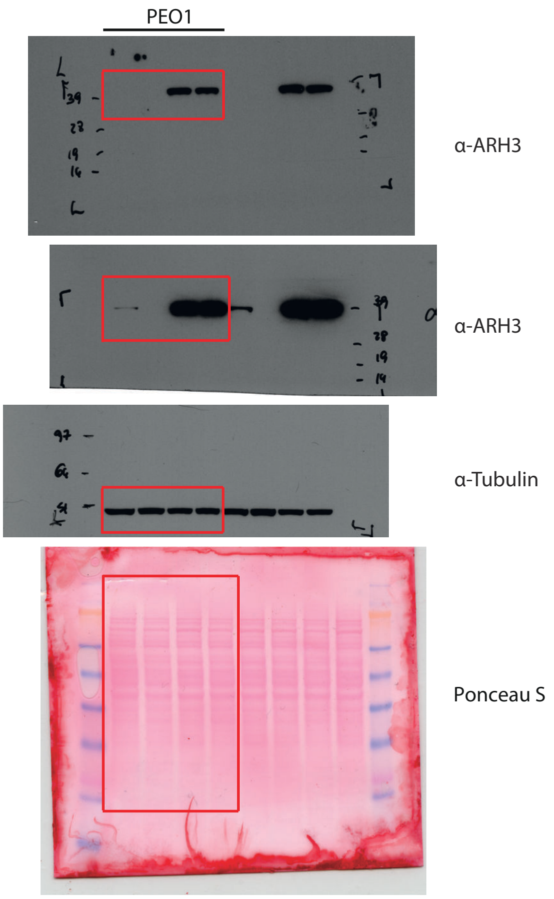

FIGURE S5A

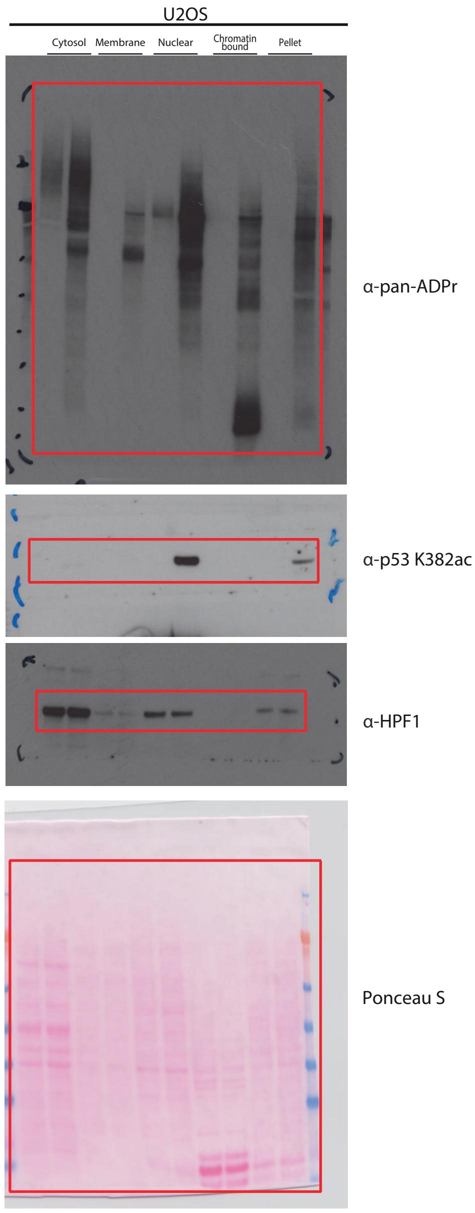

FIGURE S5B

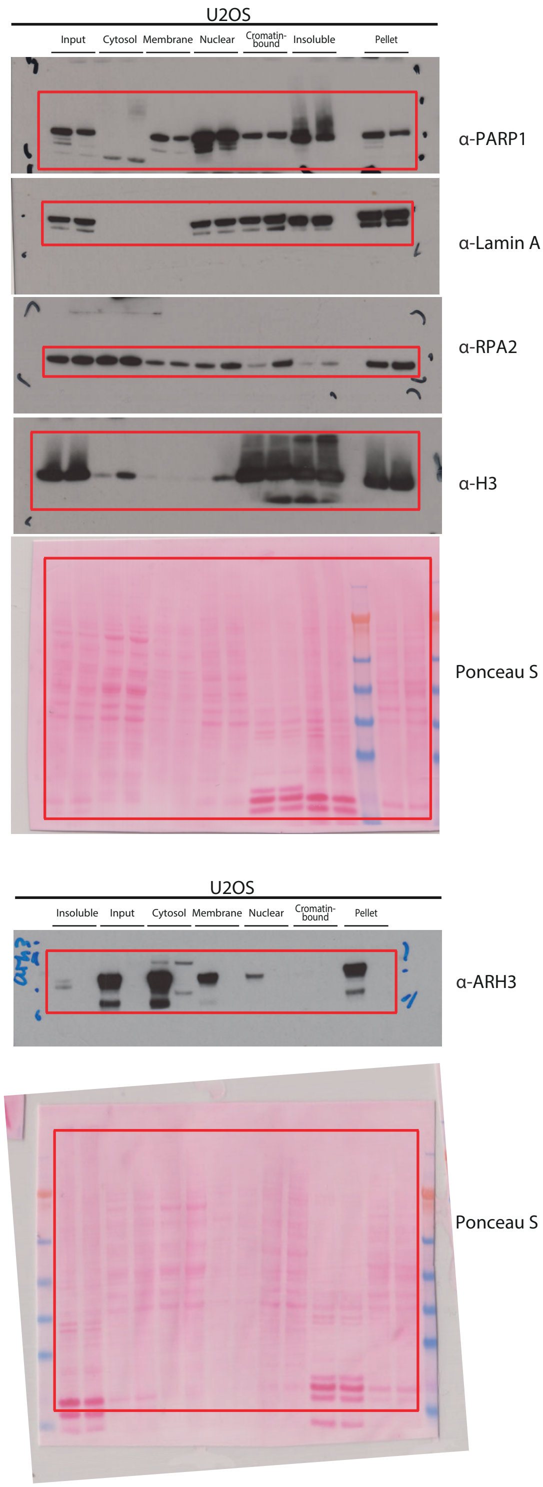

FIGURE S6C

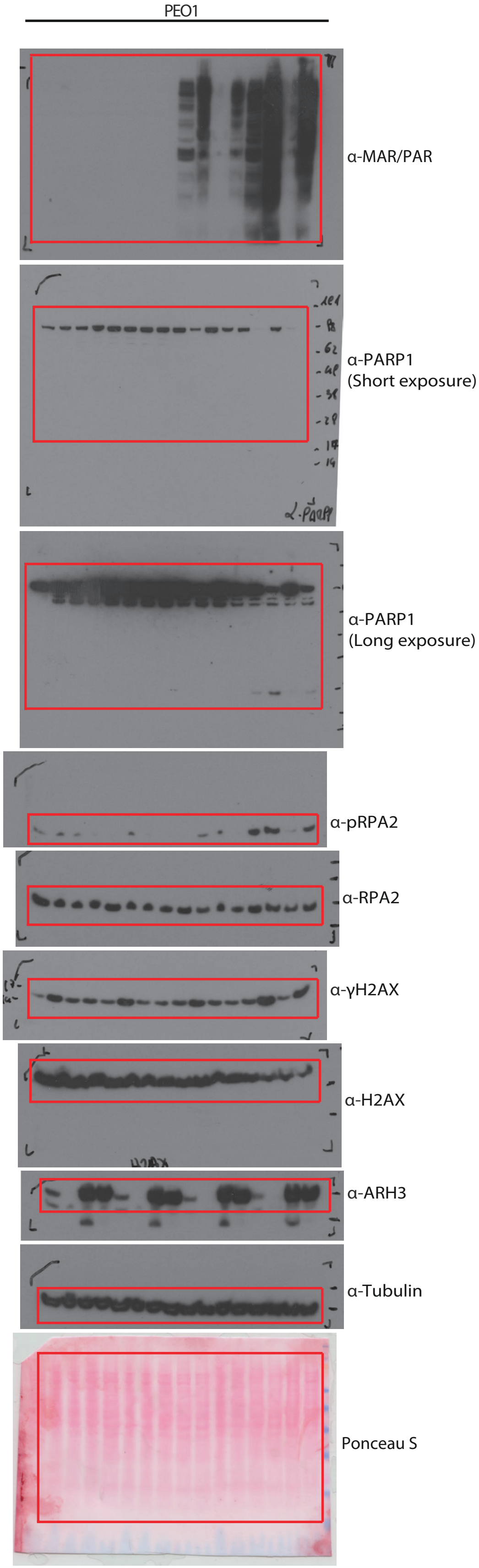

Supplement: Supplementary file 3 [file mmc3.pdf]
